# Effects of Lyse-It on endonuclease fragmentation, function and activity

**DOI:** 10.1371/journal.pone.0223008

**Published:** 2019-09-30

**Authors:** Tonya M. Santaus, Fan Zhang, Shan Li, O. Colin Stine, Chris D. Geddes

**Affiliations:** 1 Chemistry and Biochemistry Department, University of Maryland, Baltimore County, Baltimore, Maryland, United States of America; 2 Institute of Fluorescence, University of Maryland, Baltimore County, Baltimore, Maryland, United States of America; 3 Epidemiology and Public Health Department, University of Maryland School of Medicine, Baltimore, Maryland, United States of America; Meharry Medical College, UNITED STATES

## Abstract

Nucleases are enzymes that can degrade genomic DNA and RNA that decrease the accuracy of quantitative measures of those nucleic acids. Here, we study conventional heating, standard microwave irradiation, and Lyse-It, a microwave-based lysing technology, for the potential to fragment and inactivate DNA and RNA endonucleases. Lyse-It employs the use of highly focused microwave irradiation to the sample ultimately fragmenting and inactivating RNase A, RNase B, and DNase I. Nuclease size and fragmentation were determined visually and quantitatively by SDS polyacrylamide gel electrophoresis and the mini-gel Agilent 2100 Bioanalyzer system, with a weighted size calculated to depict the wide range of nuclease fragmentation. Enzyme activity assays were conducted, and the rates were calculated to determine the effect of various lysing conditions on each of the nucleases. The results shown in this paper clearly demonstrate that Lyse-It is a rapid and highly efficient way to degrade and inactivate nucleases so that nucleic acids can be retained for down-stream detection.

## Introduction

Ribonuclease A (RNase A), Ribonuclease B (RNase B), and Deoxyribonuclease I (DNase I) are three types of stable endonucleases that can contaminate RNA and DNA samples and effectively cleave a wide variety of genomic DNA and RNA prior to being detected by methods like quantitative polymerase chain reaction (qPCR). Ribonuclease A is a small 13.7 kDa protein with 124 amino acid residues, including eight cysteines that create four crosslinking disulfide bonds. The presence of the disulfide bonds, in addition to the protein’s ability to refold, lends to its immense stability. RNase A hydrolyzes the phosphodiester bond of a nucleoside 2’3’-cyclic phosphodiester of both single and double-stranded RNA.[1, 2] Like RNase A, RNase B has an identical amino acid sequence to RNase A, but it is also glycosylated by an *N*-linked oligosaccharide chain at Asn34.[3] The carbohydrate chain extends outwards from the protein in to the cytosol and can be hydrated. RNase B has the same catalytic ability as A, but it is slightly heavier due to glycosylation, measuring around 14.8 kDa.[1] The oligosaccharide chain imparts RNase B with slightly higher stabilization at a pH greater than 7.[4, 5]

Unlike RNase A and B, DNase I is a large nuclease, typically around 31 kDa and is typically characterized by five main criteria. The criteria include a preference for double-stranded DNA as a substrate, an optimum pH between 7 and 8, endonucleolytic attack, production of 5’-oligonucleotides, and the requirement of divalent cations to function. DNase I has no native activity without Mg^2+^ cofactors and is naturally inhibited by monomeric actin or when in the presence of chelating agents.[6–9]

Herein, we have investigated the degradation, fragmentation, and enzymatic activity of these three nucleases with conventional heating, standard microwave irradiation, and using a highly focused microwave irradiation based technology known as Lyse-It. Lyse-It has been reviewed in the literature as a highly efficient way to rapidly break open a wide variety of bacterial cells and subsequently fragment DNA and RNA and degrade proteins.[10–14] Lyse-It is a technology that utilizes highly focused microwave that increases the electromagnetic energy and subsequently temperature to the sample. By using gold equilateral triangles and a standard microwave, microwave power and time can be adjusted for tunable cellular lysis, intracellular component release, DNA fragmentation, and protein degradation.[12–14] We investigate the effects of conventional heating, standard microwave irradiation versus Lyse-It for their ability to fragment and inactivate three endonucleases. By utilizing Lyse-It, we can conclude that cellular lysis, DNA fragmentation, protein release and degradation [14] along with nuclease fragmentation, can occur at specific Lyse-It power and time settings, and be tuned to achieve over 98% inactivation of RNase A, RNase B, and DNase I.

## Materials and methods

### Standardization of nuclease concentrations

The three nucleases used in this paper were Ribonucleases A and B and Deoxyribonuclease I. All nucleases were purchased from Sigma Aldrich and stored at -20°C until use. The concentration of the nucleases was standardized at 6.1 ± 0.2 μM in DI water for all stock solutions. Concentrations of 11 ± 1 μM were used for the 2100 Agilent Bioanalyzer 80 Protein Kit studies. Concentrations were adjusted to 20 pM RNase A, 46 pM RNase B, and 10.5 nM DNase I for the activity studies with RNaseAlert QC v2 system (Thermo Fisher 4479769) and the DNaseAlert QC system (Thermo Fisher AM1970) respectively. The absorbance of RNase A and RNase B was determined using Beer-Lambert Law using a 0.5 cm pathlength quartz cuvette (Starna) and an extinction coefficient of 9800 M^-1^cm^-1^. The extinction coefficient for RNase B was calculated as 11760 M^-1^cm^-1^, and its absorbance was measured using a 0.5 cm pathlength quartz cuvette. The extinction coefficient for DNase I was calculated to be 34,410 cm^-1^M^-1^ as compared to 36,750cm^-1^M^-1^ reported by the Worthington Biochemical Corp.[15]

### Buffer mixtures

25 μL of the standardized nuclease concentrations described above were mixed with 25 μL of a buffer solution to create sample nuclease concentrations of 3.0 ± 0.2 μM. The final concentration of nucleases for SDS polyacrylamide gel electrophoresis (PAGE) were 3.0 ± 0.2 μM. From this concentration, nucleases were diluted down using serial dilutions to the concentrations stated in the standardization of nucleases methods section for the assay studies. The buffers that were used were 0.1 nM, 1.0 nM, 1.0 μM, 1.0 mM or 10 mM Tris-EDTA or HEPES and DI water. Each set of samples was subjected to either conventional heating or microwave irradiation both with or without Lyse-It.

### Nuclease conventional heating

A Fisher Scientific Isotemp heating block was used to conventionally heat all nucleases. RNase A and DNase I were heated at 60°C for 1 minute in DI water, Tris-EDTA and HEPES buffer concentrations, as listed above, to see if the type or concentration of buffer had an effect on the nucleases, as visualized by a single band seen by SDS PAGE. Additionally, RNase B was conventionally heated for 1 minute at temperatures between 40°C and 80°C in 1 mM Tris-EDTA or. Changing the temperature aided in the investigation of whether temperature promoted nuclease fragmentation as probed by SDS PAGE.

### Nuclease purging with argon, air, or oxygen

Nucleases were purged with argon, air, or oxygen for 10 minutes with a steady bubble per couple of seconds. A Lyse-It slide was used and enclosed with a 3 mL sample volume chamber containing 1250 μL of sample. A lid was adhered to the top of the sample chamber to contain the sample and create a sealed purging system, and to trap the purging gas. A venting needle was inserted through the sample chamber above the sample and the gas-in needle was inserted at the bottom of the sample chamber into the sample (S1 Fig). The bubble rate was monitored for the 10 minutes of purging. After 10 minutes, the venting needle was removed, and gas bubbles allowed a gaseous headspace to be created until removed a few seconds later. After purging, the samples were microwave irradiated for 60 seconds at 50% total cavity power, where the total cavity power of the microwave was 900W. Post microwave irradiation, the sample temperature was taken with a Traceable Dual Laser IR digital Thermometer. The lid was subsequently removed, and the sample was pipetted out into a 1.5 mL microcentrifuge tube. A 50 μL 4 nM solution was made from each purged microwaved solution for use with the RNaseAlert QC v2 system (Thermo Fisher Scientific AM1970) or the DNaseAlert QC system (Thermo Fisher Scientific 4479769).

### Microwave irradiation both *with* or *without* Lyse-It

One set of nuclease samples was microwaved either *with* or *without* Lyse-It for 30 seconds at either 30% or 50% total power for each buffer concentration listed above. Additionally, each nuclease was added to 1 mM Tris EDTA and microwaved at 30% power for 30, 45, 60, 90, or 135 seconds respectively, to test the effects of microwave irradiation time. It is imperative to note that the Lyse-It system employs a Frigidaire 900-Watt microwave. Note, the Lyse-It technology is not a theory driven lysing technology, even though it utilizes microwaves (see reference 10).

### Visualization of nuclease molecular size and weighted concentration through SDS-PAGE

20 μL of each sample was loaded and run against unheated controls on a 10% SDS-PAGE and stained overnight with Oriole dye and DI water was used to wash off any excess Oriole stain. The gel was then imaged using a Bio-Rad GelDoc EZ Imager. The intensity of each visible band was analyzed against a standard *Pre*-nuclease band to determine if degradation or fragmentation of the nuclease had occurred. The *Pre*-nuclease band intensity and *Pre*-nuclease activity were obtained through the suspension of nuclease in buffer without any further analysis.

### Nuclease fragmentation and weighted size analysis using the Agilent 2100 Bioanalyzer

In order to analyze and ultimately quantify small nuclease fragments post lysing, RNase A, RNase B, and DNase I samples were run on an Agilent Bioanalyzer 2100 system utilizing the Agilent Protein 80 Kit. The Bioanalyzer analysis was performed at the Institute of Marine and Environmental Technology BioAnalytical Services Lab in Baltimore, MD, USA. Because the nuclease concentrations used coupled with the purging conditions and microwave power and times employed, smaller nuclease fragments were not readily seen on the SDS PAGE; a more sensitive detection method was used to see and quantitate nuclease fragmentation, i.e. the 2100 Bioanalyzer system. Only one trial on the Agilent 2100 Bioanalyzer system was performed on each of the nucleases under the varying conditions to show that each of the nucleases were being fragmented into various sizes that could now be visualized on SDS PAGE. This data is concurrent with previous data that shows that bacterial DNA is also fragmented in to various sizes under a wide variety of conditions.[12–14] To aid in the fragment analysis, a mathematical weighted size (${\overset{-}{y}}_{1\to n}$) was calculated per trial as shown in Eqs 1→ 2, where *n*_*0*_ is the first peak detected and *n* is the final peak reported from the Bioanalyzer data. For these equations, *y*_*i*_ *wa*s the reported fragment size at peak *n*_*0*_ *≤ i ≤ n*, *R*_*i*_ is the relative concentration reported for each fragment, and *x*_*i*_ was the calculated weight factor where 1 = ∑_*i*_ *x*_*i*_.

$${\overset{-}{y}}_{n_{0}\to n}=\sum_{i=n_{0}}^{i=n}x_{i}y_{i}$$ (1)

$$x_{i}=\frac{R_{i}}{\sum_{i=n_{0}}^{i=n}R_{i}}$$ (2)

The weighted fragment sizes for each trial were then averaged, and the standard deviation calculated. Weighted averages demonstrating larger relative standard deviation values correspond to a larger distribution of observed fragmentation within the sample set.

### Measured values of nuclease activity using RNaseAlert and DNaseAlert QC systems

Nuclease activity studies were performed using RNaseAlert QC v2 and DNaseAlert QC systems from Thermo Fisher Scientific. To be brief, the two assays work in a FRET/PET manner where a fluorophore is connected to a DNA or RNA strand on one end and a respective quencher tethered on the other end. This fluorophore/quencher strand “pair” is known as the substrate. When the substrate and an enzyme, like RNase A/B or DNase I come in contact, the nuclease readily cleaves the substrate allowing for the fluorophore to be released and to subsequently fluoresce upon excitation (S2 Fig). Thus, as more substrate is cleaved, the more fluorescence is detected and can be monitored in real-time using a standard fluorometer. It is important to understand that the fluorescent intensity measures nuclease activity, i.e. as fluorescent intensity increases, the greater the activity of the nuclease. The rate is then shown as fluorescent intensity over time. 4 μL or 9.98 μL of 4 nM RNase A or RNase B respectively, was added to 776 or 771 μL DI water, 10 μL RNaseAlert Buffer, and 10 μL v2 fluorescent substrate. 4 μM DNase I (2 μL) was added to 778 μL nuclease free water, 10 μL RNaseAlert Buffer, and 10 μL fluorescent substrate. For both assays, the final sample volume was 800 μL. A 0.5 cm quartz cuvette (Starna) with a max volume of 800 μL was used for all fluorescent kinetic runs performed on a Horiba Fluoromax 4 Fluorometer with FluorEssence software. For RNase A and RNase B, the following parameters were set—λ_ex_: 490 nm, λ_em_: 520 nm, collections of 0.1–0.4 every 3 seconds, λ_ex_ and λ_em_ slits of 1 nm, with a total of 100 collections. For the DNaseAlert QC assay the following parameters were used—λ_ex_: 535 nm, λ_em_: 556 nm, collections of 0.1–0.4 every 3.5 seconds, λ_ex_ and λ_em_ slits of 1 nm, with a total of 100 collections. Blank measurements for both the RNase and DNase Alert assays were performed without nuclease added for 10 collections with the same parameters above, prior to the addition of nuclease.

### qPCR of *Vibrio cholerae* DNA after exposure to intact and microwave irradiated DNase I with and without Lyse-It

Stock DNase I (2.70U/μL) was diluted to 0.1U/μL. Three tubes were made where there was 1U of DNase I in a total volume of 50μL One of the tubes was DNase I not microwaved and serves as the intact DNase I control. From the second tube of 1U DNase I, all 50μL were microwave irradiated without Lyse-It for 60 seconds at 50% power. Finally, the last tube containing 50μL 1U DNase I was microwave irradiated at 50% power for 60 seconds with Lyse-It. 0.5U of each of the 3 different trials were incubated with 1μg of *V*. *cholerae* DNA. Incubation was performed in a 37°C heating block for 10 minutes. The *V*. *cholerae* DNA/ DNase I reactions were stopped by increasing the heating block temperature to 100°C and heating the reaction for 5 minutes. qPCR was then run on the samples using an ABI QuantStudio 3 qPCR with the following *V*. *cholerae* primers. hlyA forward primer: 5’-ATCGTCAGTTTGGAGCCAGT-3’ and hlyA reverse primer 5’-TCGATGCGTTAAACACGAAG-3’.

## Results

### Modest conventional heating and buffers do not affect RNase A, RNase B, or DNase I fragmentation or aggregation

Prior to studying the effects of lysing on nuclease activity, it was imperative to investigate nuclease size band intensity via SDS PAGE to ascertain if there were any affects due to buffer at 60°C or also an increase in temperature. 60°C was used as the conventional heating temperature because of the microwave irradiation power and times used, which also generated an average upper sample temperature of 60°C ± 5°C. RNase A and DNase I were conventionally heated at 60°C for 1 minute in various buffer concentrations (S3A Fig) to see the effects on the RNA/ DNA endonuclease in varying concentrations. Additionally, RNase B was conventionally heated at varying temperatures to determine if an increasing temperature reduced the ability of the Oriole stain to bind to the nuclease (S3B Fig). Normalization was performed where the band intensity of the *Pre* (not conventionally heated) nuclease was set to 1. Each band after Pre was divided by the *Pre*-band intensity. For RNase A/B and DNase I, the buffer concentration for Tris-EDTA and HEPES as well as temperature did not affect the band intensity of the nucleases. Note that for all RNase A/B and DNase I SDS PAGE there was only 1 band (the nuclease size) that was visible on the Oriole stained gels. Only band intensity could be distinguished.

### Statistically significant band intensity differences occur when nucleases were microwave irradiated with Lyse-It with increasing powers and increasing irradiation times

Following conventional heating, we investigated the effect that Lyse-It had with increasing microwave power for a constant 30 seconds on nuclease band intensity in Tris-EDTA, HEPES, and DI water. The average nuclease band intensity for each buffer was taken across all concentrations of buffer, as concentration did not influence band intensity. For the DI water average, different samples of nuclease in DI water and microwave irradiated were loaded into three separate lanes on the SDS PAGE. The reference band intensity used was the *Pre*-band as the nuclease was only suspended in DI water without any further processing. A line graph of RNase A in each of the buffers was constructed to see the normalized band intensity (S4A Fig). It was found that for RNase A there were statistical differences (p<0.05) between Tris-EDTA and DI water and HEPES and DI water for 50% total microwave power. At 30% total microwave power, only HEPES to DI water was significant (p<0.05) (S4B Fig). Additionally, DI water was the only buffer to show a significant statistical difference (p<0.05) between 30% power irradiation and 50% power irradiation (S4C Fig). At both 30% and 50% powers, there were no statistical differences between Tris-EDTA and HEPES buffers.

RNase B displayed similar results to RNase A (S5A Fig) such that there were statistical differences (p<0.05) between Tris-EDTA and DI water and Tris-EDTA and HEPES at 50% total cavity power. However, there was only a statistical difference (p<0.05) between Tris-EDTA and HEPES at 30% total cavity power (S5B Fig). However, there were no statistical differences when keeping the buffer constant but changing the microwave power (S5 Fig).

For DNase I, there were statistical differences (p<0.05) seen in band intensity when all buffers were kept constant and only the microwave power was changed (S6A and S6C Fig). On the contrary, to the ribonucleases, there were only statistical differences (p<0.05) at 30% power between Tris-EDTA and DI water and HEPES and DI water (S6B Fig).

**Fig 1:**
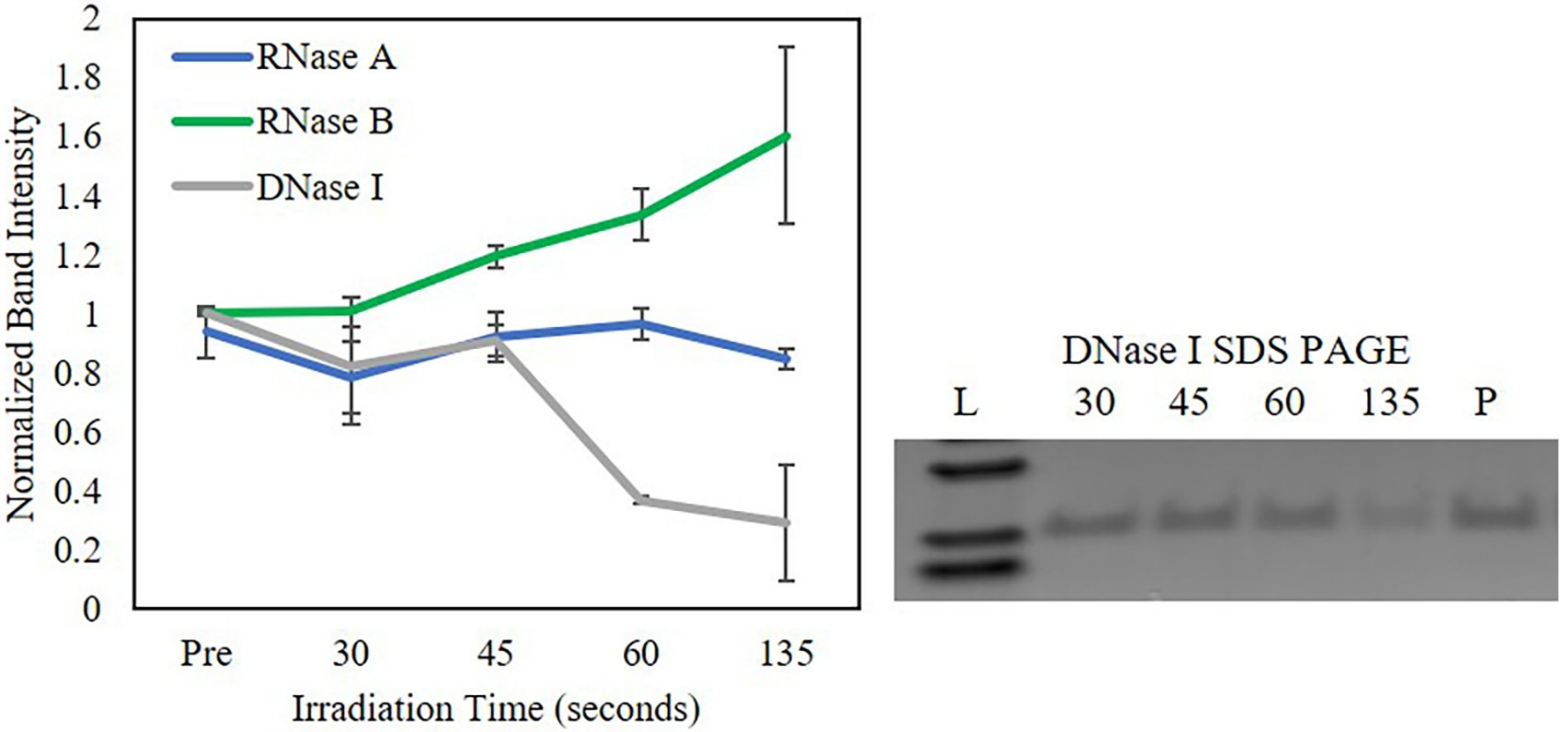
SDS PAGE band intensities for RNase A, RNase B, and DNase I in 1 mM Tris-EDTA post 30% microwave irradiation with Lyse-It. A representative SDS PAGE for DNase I is provided were a decrease in band intensity is seen as microwave irradiation time increases. For RNase A and B, the band intensities did not decrease as irradiation time increased and thus, there were no statistical differences for RNase A and B.

In addition to microwave power, microwave irradiation time was also investigated keeping both the buffer (1 mM Tris-EDTA) and Lyse-It power constant (30%). Band intensities were normalized to 1, where 1 was equal to the *Pre*-band intensity for each nuclease (Fig 1A). It was only seen for DNase I that an increasing irradiation time reduced the band intensity for times greater than 60 seconds (p<0.05) (Fig 1B). We attribute the increase in RNase B increase in band intensity as irradiation time increased do to gel abnormalities such as a curved gel and the stacking portion of the gel making a non-compressed band (S7 Fig).

Although there were some band intensity differences that could be seen in the SDS PAGE gels with respect to microwave power and time, nuclease fragmentation was not evident confirming that the band intensity decrease was likely due to nuclease degradation. Therefore, we utilized a more sensitive technique, namely the Agilent 2100 Bioanalyzer mini-gel system, for protein size and fragmentation determination.

### As oxygen concentration increased within the nuclease samples, more fragments were seen

**Fig 2:**
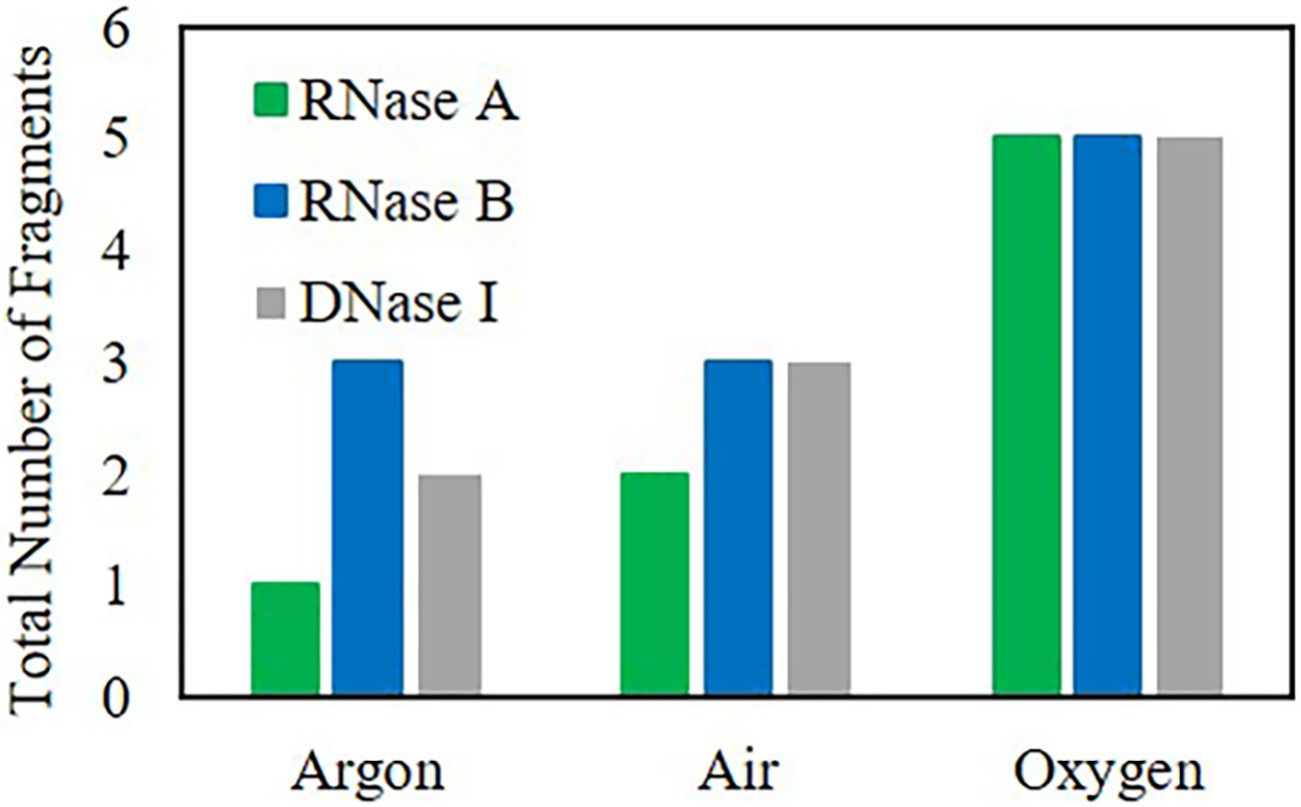
Total number of fragments observed for RNase A, RNase B, and DNase I lysed with Lyse-It at 30% power, 60 seconds using the Agilent 2100 Bioanalyzer system. As oxygen content increases, the number of nuclease fragments also increases.

It is well-known that reactive oxygen species (ROS) and reactive nitrogen species (RNS) affect cells and mechanisms within cells, oxidize various molecular species, and have the ability to cut, mutate, fragment, and degrade nucleic acid species.[16–22] Additionally, it has been shown that Lyse-It generates more reactive oxygen species, in particular singlet oxygen, hydroxyl radicals, and superoxide anion radicals as oxygen content to the sample increases as well as microwave irradiation power.[23, 24] Therefore, we investigated the effects of increasing the oxygen content within the nuclease suspensions prior to microwave irradiation with Lyse-It. To be able to see the fragments, the Agilent 2100 Bioanalyzer system with the Protein 80 kit was used. The three nucleases were purged with argon, air, or oxygen for 10 minutes and then immediately microwaved with Lyse-It at 30% power for 60 seconds. It was shown for all nucleases that as the oxygen content increased, the number of fragments also increased (Fig 2), which is consistent with previous reports of ROS-based fragmentation.[20, 22, 25–28] To achieve a more complete analysis of the fragments, the total number of fragments from Fig 2 were subsequently broken down by kDa size ranges and are reported in S1 Table. To be brief, the Bioanalyzer displayed sizes that were consistent with dimers for RNase A (approximately 27 kDa) and RNase B (approximately 29 kDa) and the monomer form of DNase I (approximately 31 kDa).

### As microwave power and time increase, the number of nuclease fragments increases

**Fig 3:**
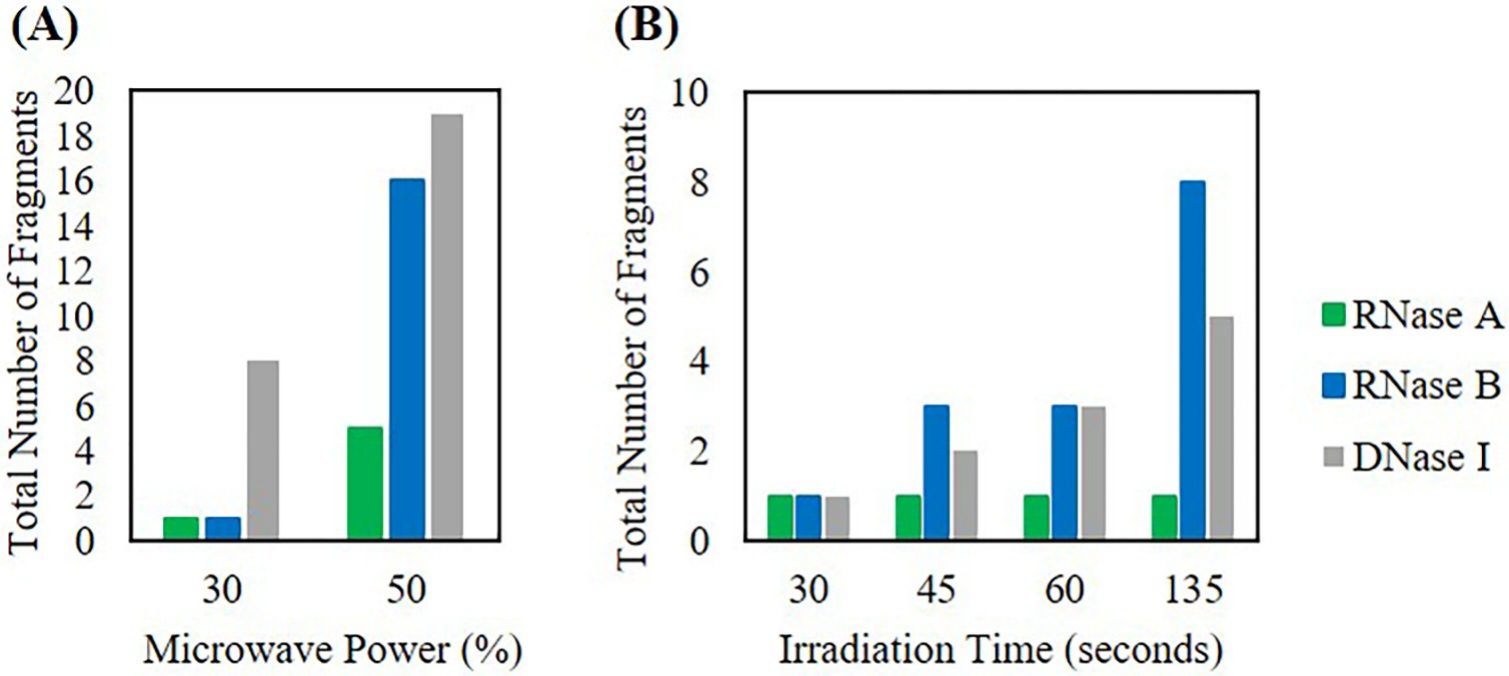
Total number of protein fragments for nucleases microwave irradiated with Lyse-It in DI water analyzed using the 2100 Bioanalyzer. Increasing the microwave irradiation power with Lyse-It **(A)** and Lyse-It irradiation time **(B)**. As microwave power or irradiation time increases, the number of nuclease fragments also increases.

Following nuclease fragmentation with increasing oxygen sample content above, we additionally investigated nuclease fragmentation following Lyse-It at 30% and 50% power at 60 seconds and 30% power with an increasing irradiation time (Fig 3). Consistent with previously reported work, as microwave power is increased, the size of DNA fragments decreased demonstrating that large genomic DNA was being fragmented down into smaller sized pieces.[12, 14] This same concept holds true when it is applied to nucleases. As the microwave power increased, the number of fragments increased (Fig 3A). Additionally, as microwave power was kept constant and irradiation time increased, the number of nuclease fragments increased (Fig 3B). As microwave power increased or as irradiation time increased for each nuclease, the number of fragments found within various size ranges (kDa) can be found in S2 and S3 Tables respectively. It is important to note that fragments larger than the monomer of DNase I were larger than the reported kDa size. We think this is due to rearrangement or aggregation, with DNase I forming dimers or larger aggregates. This is also thought to be similar for RNase A or B for fragments that are larger than the reported monomer or dimer sizes. Even though we were able to see fragments from a variety of conditions, the increase in oxygen content, microwave power or time both increased and decreased the size of fragments; therefore, we considered a weighted fragment size analysis and standard deviation for each of the conditions to further understand this.

### As oxygen content or microwave power or time increased, the overall weighted size decreased while the standard deviation became larger

**Fig 4:**
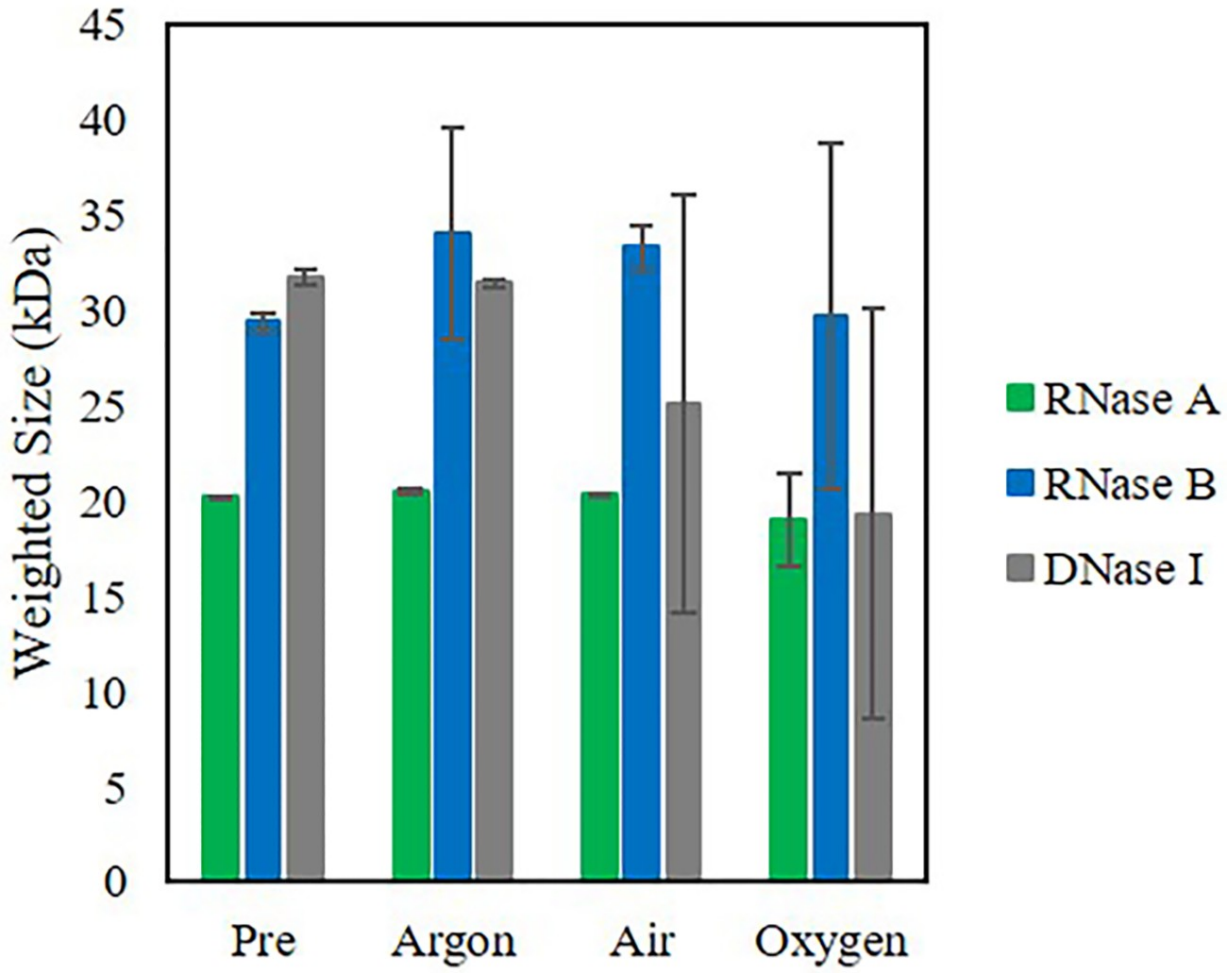
Weighted size (kDa) of RNase A, RNase B, and DNase I. Weighted size post purging and subsequent microwave irradiation of 30% power, 60 seconds. In general, as oxygen content increases, the overall weighted nuclease size decreases and the error in the size becomes larger. This in indicative of an increasing number of varying size fragments with an overall degraded nuclease. Pre = No microwave irradiation.

The weighted size analysis demonstrates that as oxygen content increased, the nuclease size subsequently decreased from the *Pre*-reported size, the control sample. The standard error in the size substantially increased (Fig 4) which is counter-intuitive as normally small errors are reported to confirm a small measured size. Here, the error was large as there was not only one quantitative size found but numerous sizes of the nuclease fragments. This standard error analysis was also applied to the other conditions investigated.

**Fig 5:**
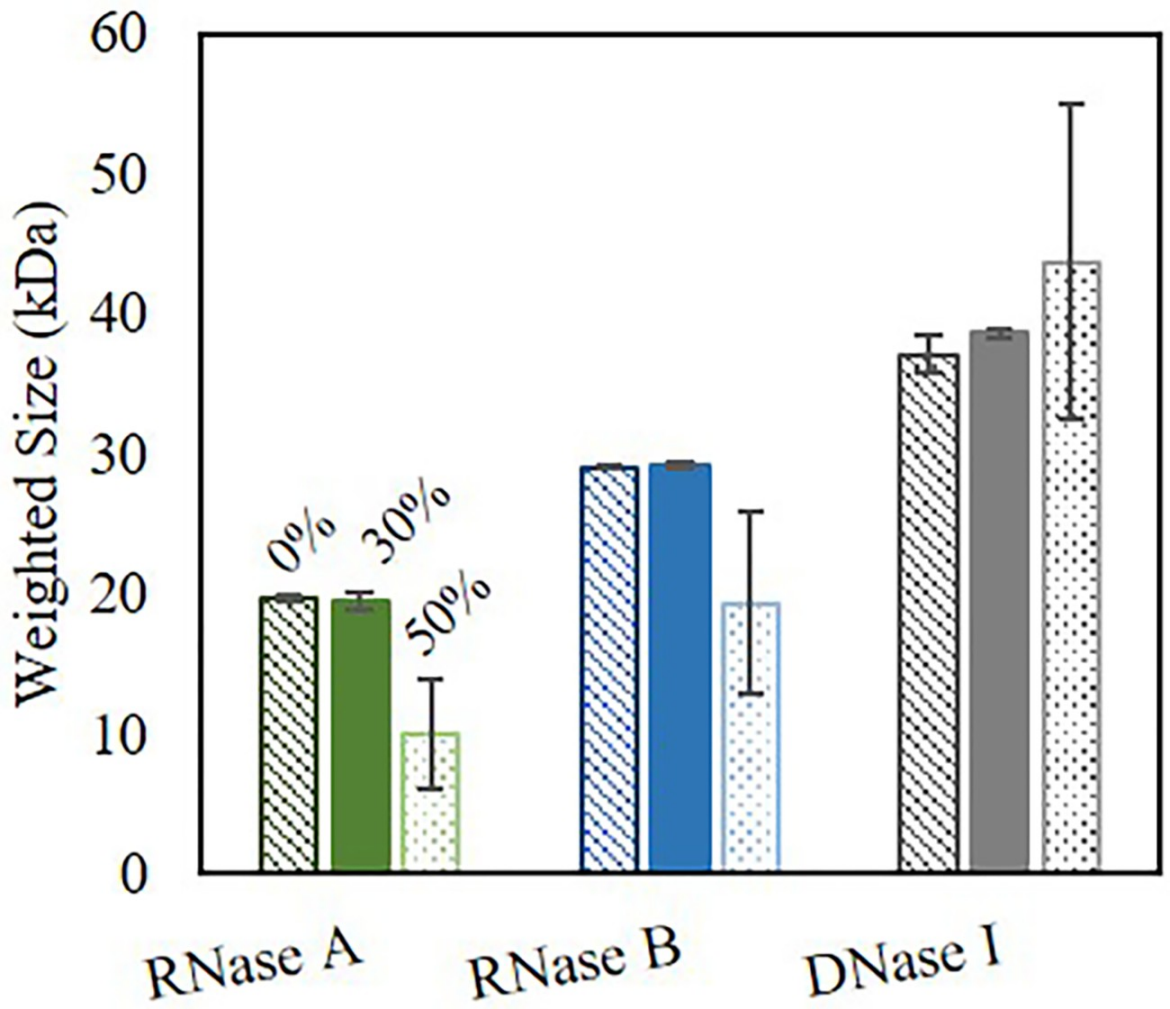
Weighted size (kDa) of RNase A, RNase B, and DNase I. Weighted size post microwave irradiation of 30% and 50% power, 60 seconds as compared to *pre (0%)*. In general, as microwave power increases, the overall weighted nuclease size decreases and the error in the size becomes larger. This in indicative of an increasing number of varying size fragments with an overall degraded nuclease.

**Fig 6:**
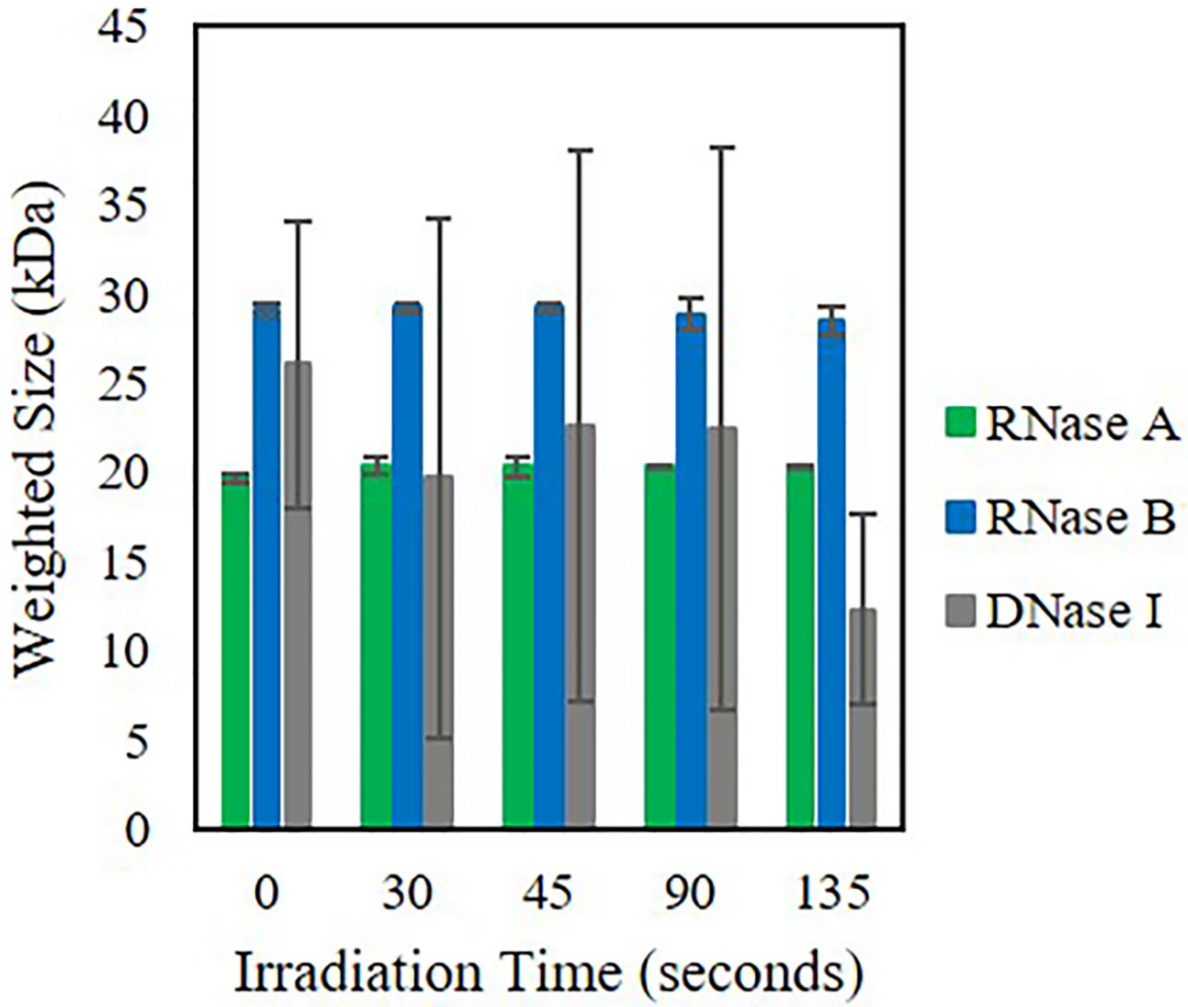
Weighted size (kDa) of RNase A, RNase B, and DNase I. Weighted size post microwave irradiation at 30% power, 60 seconds. In general, as irradiation time increases, the overall weighted nuclease size decreases and the error in the size becomes larger. This in indicative of an increasing number of varying size fragments with an overall degraded nuclease. Pre = No microwave irradiation.

Following the weighted sizes from the increasing oxygen content above, we studied the effects of an increase in total microwave cavity power (30% to 50% for 60 seconds) and irradiation time (30 through 135 seconds). In terms of band intensity, there were some visual differences for the three nucleases, but within the SDS PAGE gel, nuclease weighted size or various fragments could not be seen or determined. Just as oxygen content increased and the number of nuclease fragments subsequently increased, as microwave power increased, in general the weighted nuclease size decreased (Fig 5). DNase I was the only nuclease where the weighted size was slightly higher than that of the reported size. We attributed this to a potential increase in aggregates or DNase I dimers. On the contrary to the increase in microwave power, as irradiation time increased, RNase A and RNase B weighted size was unaffected (Fig 6). However, for DNase I, the weighted size decreased and the error was significant for all irradiation times, indicative of a large number of fragments. This suggests that DNase I is more susceptible to fragmentation then either RNase A or RNase B.

Thus far, we have investigated nuclease band intensity, nuclease fragments, and determined a weighted size analysis of the fragments for the three nucleases. However, none of these methods analyzed the functional activity of the nucleases. Therefore, under the same conditions as described above, the nucleases were tested with the RNaseAlert and DNaseAlert enzyme activity assays.

### Despite nucleases appearing thermally stable, the activity decreases as the heating temperature increases

**Fig 7:**
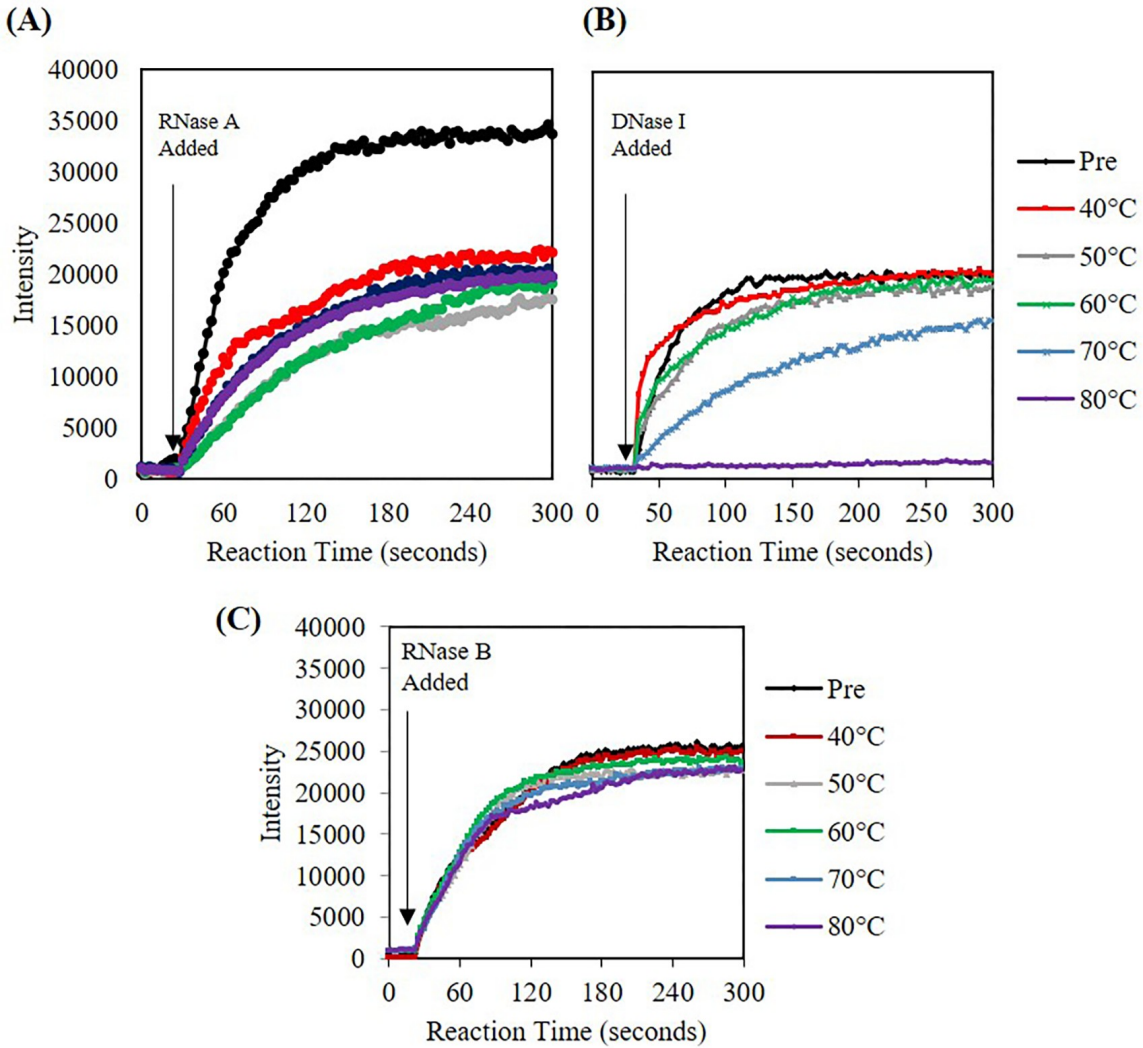
Fluorescent intensity at 520 nm (RNase A) and 556 nm (DNase I) and 520 nm (RNase B) versus time for conventionally heated (1 minute) nucleases. **(A)** RNase A **(B)** DNase I **(C)** RNase B. For RNase A and DNase I, as temperature increases, the activity of the nucleases decreases. For RNase B, the rate is statistically different at 70°C and 80°C.

The activity of the nuclease showed a temperature dependent decrease in enzyme activity. After conventionally heating samples at various temperatures for 1 minute, the samples were cooled down to room temperature prior to use with the activity assay. This cooling allowed for only the effects of conventional heating on the nuclease to be examined, i.e. not the temperature effects on fluorescence. RNase A showed a decrease in activity where the kinetic rate plateaued after 40°C (Fig 7A). However, for DNase I, a significant decrease in rate is seen after 60°C (Fig 7B). For RNase B, as temperature increased, there was no significant difference in activity until temperatures greater than 60°C (Fig 7C). Kinetic rates for each of the conditions were calculated using Excels mathematical calculation LINEST where only the linear region of the kinetic fluorescence response was considered. RNase B rate results from conventional heating are shown in Table 1. It was only until temperatures reached 80°C that the nuclease activity was diminished by ~25%. The kinetic rates for RNase A and DNase I can be found in S4 Table. In general, for RNase A, an increase in temperature decreased the activity, but remained at a constant 50% active without further loss of activity. For DNase I, there was a significant temperature activity dependence after 60°C and by 80°C the nuclease was almost over 98% inactive which is consistent with other literature reports.[9]

**Table 1:** RNase B rates and percentage still active post conventional heating for 1 minute between 40°C and 80°C.

*Conventional Heating -Temperature (°C)*	Rate (Fluorescent Intensity per Second)	Nuclease Percentage Still Active
	*RNase B*	
*Pre (RT)*	136.7 ± 2.1	100%
*40*	136.7 ± 2.1	100%
*50*	143.6 ± 6.9	100%
*60*	130.9 ± 6.8	95.7%
*70*	120.6 ± 6.6	86.3%
*80*	103.0 ± 6.9	75.3%

### Buffer and magnesium content can affect nuclease activity

**Fig 8:**
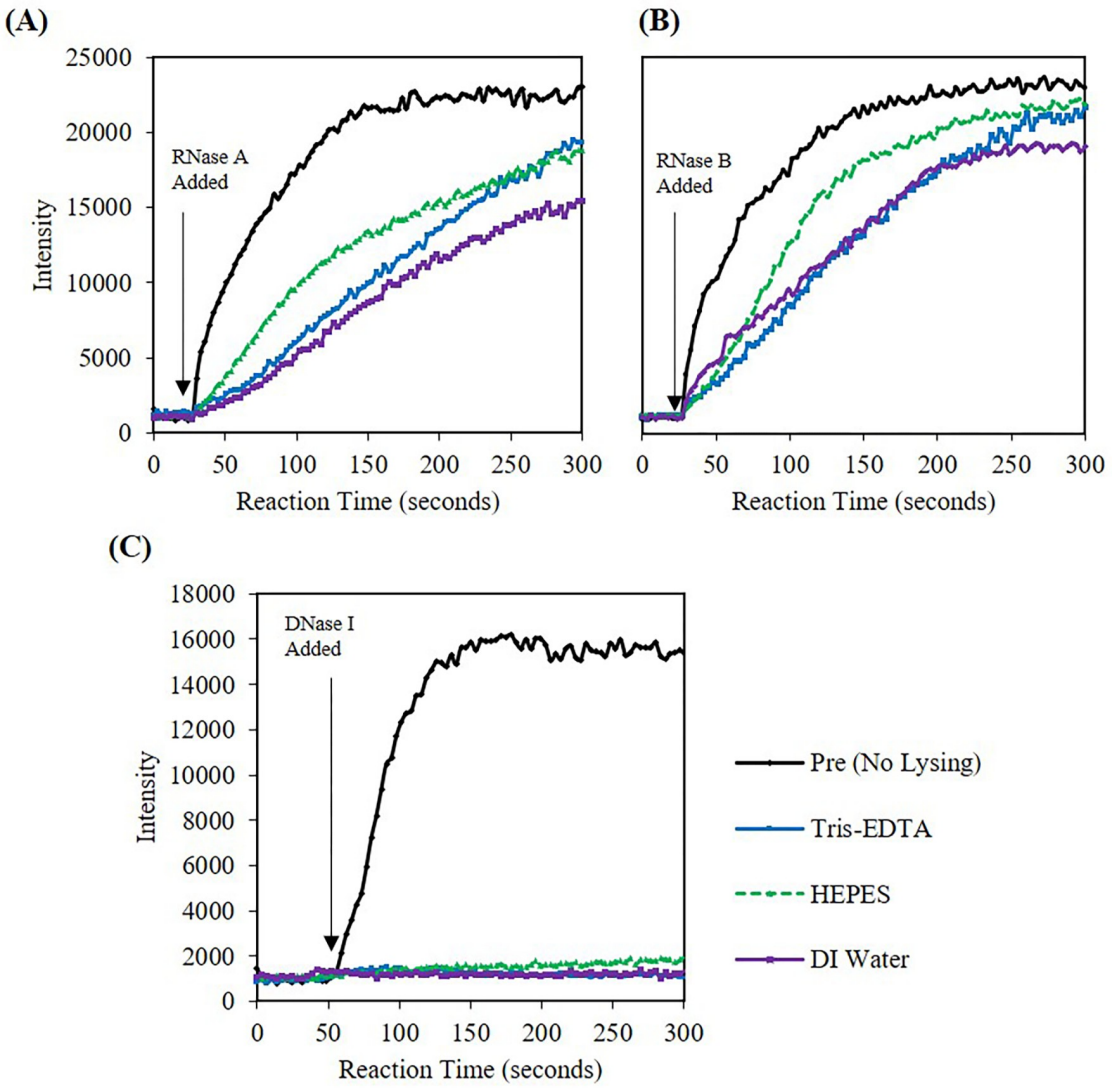
Fluorescent intensity at 520 nm (RNase A/B) and 556 nm (DNase I) versus time for nucleases in 1 mM buffers lysed with Lyse-It at 50% power for 60 seconds (A) 20 pM RNase A (B) 46 pM RNase B, (C) 10.5 nM DNase I. In general, HEPES buffer was seen to protect the nuclease from becoming more inactive as compared to Tris-EDTA or DI water.

We studied whether Tris-EDTA, HEPES or DI water had an influence on nuclease activity. Nucleases were subsequently microwave irradiated with Lyse-It for 60 seconds at 50% power. Kinetic fluorescent activity analysis for the three nucleases were obtained (Fig 8) and the corresponding rates calculated (S5 Table). In all nuclease cases, HEPES buffer showed some level of protection of the enzyme activity. It is well-known that nuclease activity, especially for DNase I, can be inhibited by metal chelating agents like EDTA.[9] Though not fully understood from these experiments, we speculated that HEPES was acting like a protective agent for the nuclease against degradation and or fragmentation and subsequent activity. Thus, as expected, the activity of the nuclease decreased in the presence of Tris-EDTA and DI water and subsequent 50% power, 60 seconds microwave irradiation with Lyse-It. Additionally, DI water was the most ineffective in nuclease activity protection as the rates were substantially lower as compared to both Tris-EDTA and HEPES buffers.

### Lyse-It significantly decreased nuclease activity compared to standard microwave irradiation

**Fig 9:**
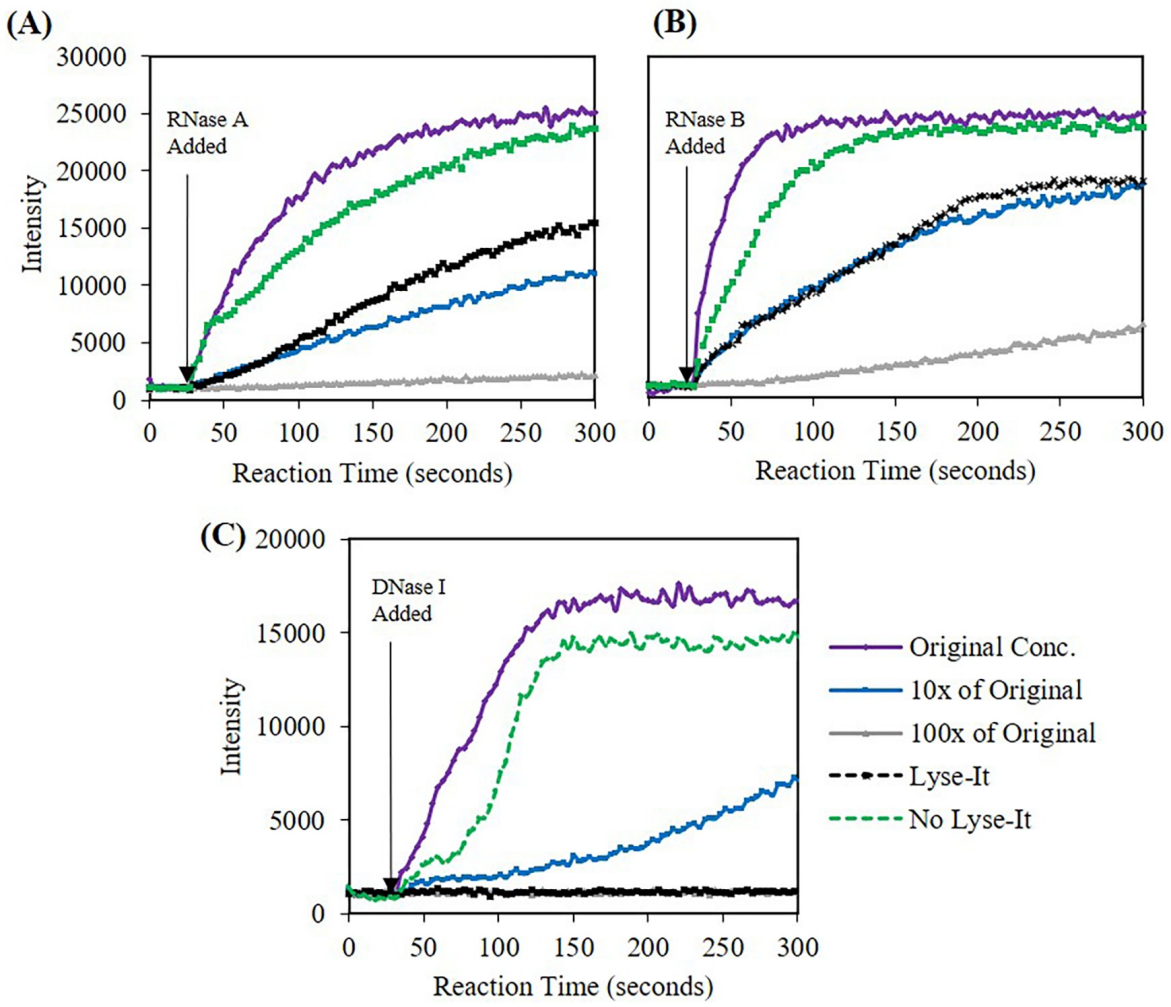
Fluorescent intensity at 520 nm (RNase A/B) and 556 nm (DNase I) versus time for nucleases in DI water lysed with and without Lyse-It at 50% power for 60 seconds. The concentration of lysing was undertaken with the highest concentration of nuclease. Each *Pre* was a 10-fold dilution from the starting concentration. **(A)** 20 pM starting RNase A **(B)** 46 pM starting RNase B, **(C)** starting 10.5 nM DNase I. When Lyse-It is used, the activity of the nuclease is significantly reduced as compared to without the use of Lyse-It.

To ascertain if Lyse-It has a similar effect on nucleases that it has on both DNA and other proteins, [12–14, 23] a dilution study of *Pre* nuclease aided in determining the degree of inactivity that Lyse-It inflicted on the nucleases. Starting with 20 pM RNase A, 46 pM RNase B, and 10.5 nM DNase I, the nucleases were diluted by 10 and 100-fold and tested with their respective activity assay (Fig 9). As the concentration of the nuclease decreased by 10-fold or 100-fold, the activity did not decrease by the same factor. This could be due to the rate fitting parameters performed in this paper or through diffusional hindrance from the suspension of the nuclease in glycerol. When comparing Lyse-It to no Lyse-It, the activity rate significantly (p<0.05) decreased over 70% from the *Pre* nuclease in suspension (S6 Table). As for standard microwave irradiation without Lyse-It over 60% of the nuclease activity remained. This confirmed that Lyse-It has a much stronger effect on diminishing nuclease activity as compared to simply microwaving without Lyse-It and to conventional heating alone.

### Increasing microwave irradiation time in combination with Lyse-It decreased nuclease activity

**Fig 10:**
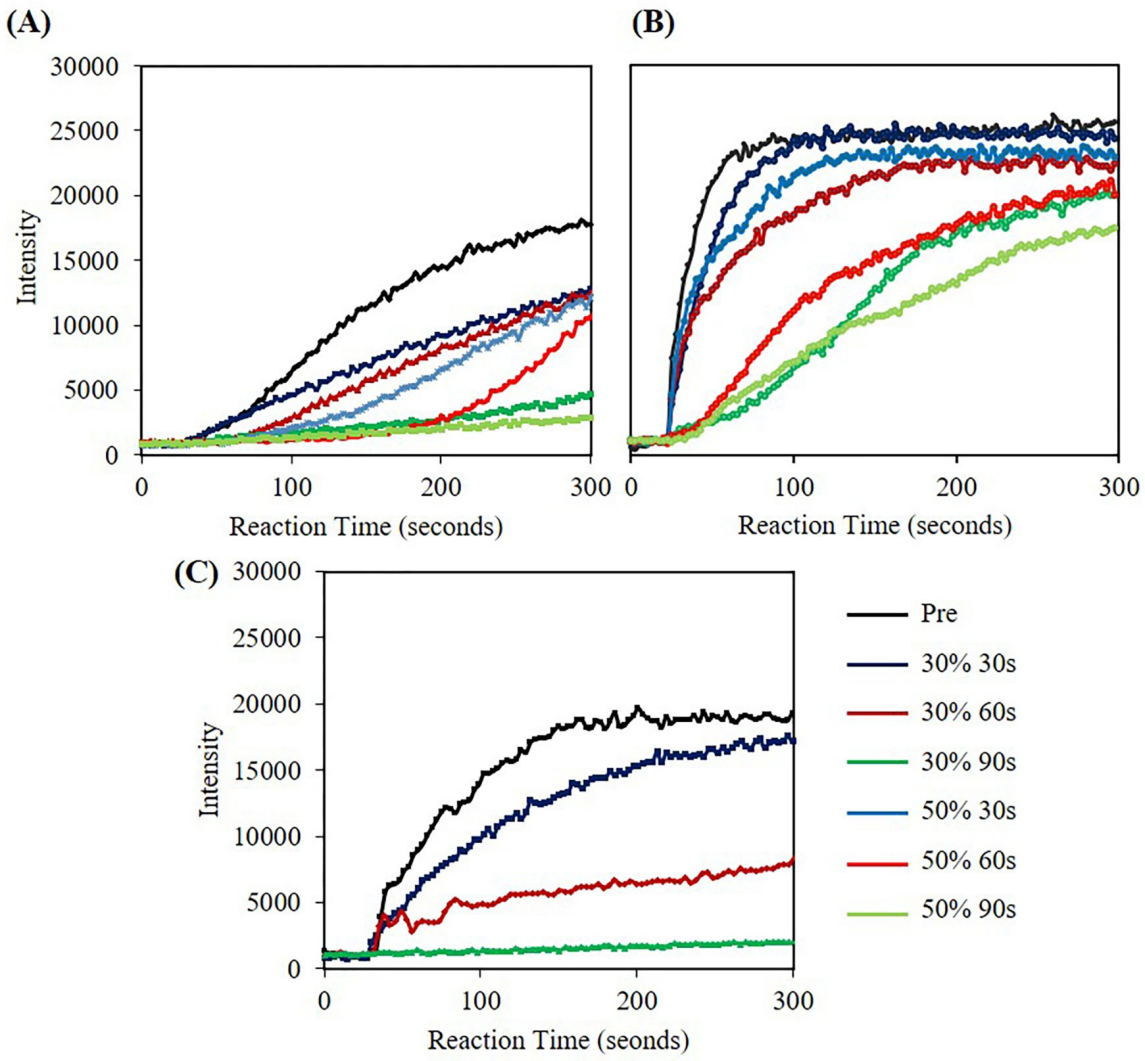
Fluorescent intensity at 520 nm (RNase A/B) and 556 nm (DNase I) versus time for nucleases in DI water lysed with Lyse-It at 30% and 50% power for varying time. *Pre* is the nuclease suspension without microwave irradiation. **(A)** 20 pM RNase A **(B)** 46 pM RNase B, **(C)** 10.5 nM DNase I. As microwave power and time increase, nuclease activity decreases.

To further investigate microwave effects on the nucleases, we looked at the activity when nucleases were subjected to constant microwave powers but now changing the irradiation times (Fig 10). In both 30% and 50% power cases, as irradiation time increased, the activity of the nuclease decreased, where RNase B is shown in Table 2 and both RNase A and DNase I in S7 Table. The 50% power series for DNase I was not performed because at 50% microwave power, the nuclease was already over 98% efficiently inactivated. At this juncture, it is important to connect back and compare microwave power versus fragmentation to the subsequent activity rate. At 50% power the number of fragments increased significantly and thus as seen with the activity rate, as the microwave power increases, the activity rate decreases. Additionally, in this experiment we were able to analyze overall microwave energy (i.e. microwave power and time) as a function of the decrease in nuclease activity. 30% microwave power for 30, 60 and 90 seconds is analogous to 8.1, 16.2, and 24.3 kJ respectively. For 50% microwave power with the same time points, the energy was 13.5, 27.0, and 40.5 kJ. This increase in energy results in a greater inactivation of the nucleases. This was particularly evident in Fig 10B with RNase B where the power and time were altered to demonstrate the increase in energy and subsequent decrease in nuclease activity. At 50% microwave power for RNase, this energy to nuclease relationship activity inactivation holds true.

**Table 2:** RNase B rates and percentage still active post Lyse-It at 30% and 50% power, varying the time. (Pre = no irradiation).

*Nuclease Irradiation Time (seconds)*	30% Microwave Power Rate (Fluorescent Intensity per Second)	50% Microwave Power Rate (Fluorescent Intensity per Second)
	*RNase B*	
*Pre*	452.0 ± 24.53 (100%)	
*30 seconds*	358.3 ± 17.15 (79%)	242.7 ± 22.87 (54%)
*60 seconds*	208.2 ± 13.84 (46%)	120.6 ± 5.87 (26%)
*90 seconds*	122.21 ± 3.73 (27%)	64.75 ± 0.83 (14%)

### Higher microwave powers and longer times, and thus energy, are required to 99% inactivate RNase A/B. Only 50% power 60 seconds (27kJ) was required to 99% inactivate DNase I

**Fig 11:**
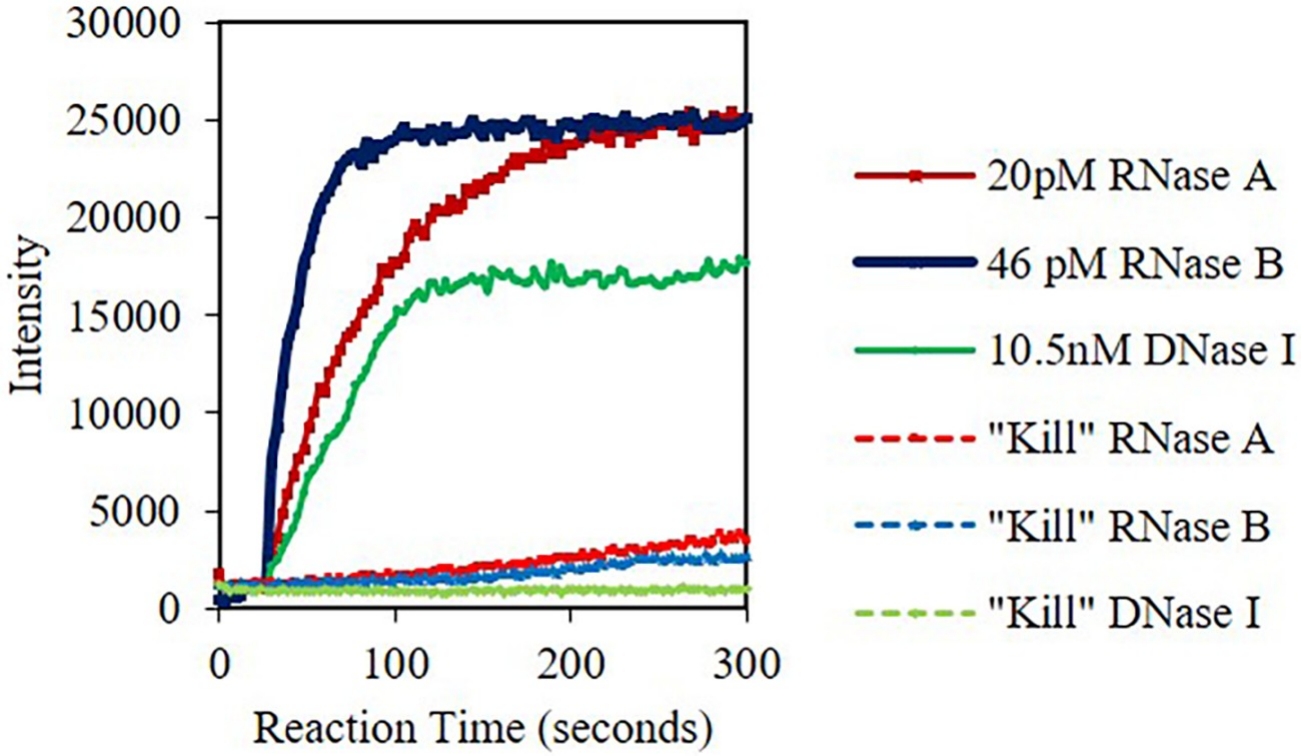
Fluorescent intensity at 520 nm (RNase A/B) and 556 nm (DNase I) versus time for nucleases post Lyse-It. RNase A and B 98% inactivation was achieved through 3 irradiations with Lyse-It of 70% power, 105 seconds. DNase I was 99% inactivated at 50% power for 60 seconds with Lyse-It.

One of the most important studies performed in this paper was the ability to inactivate the nuclease and prevent them from degrading genomic DNA and RNA. “Kill” studies were subsequently performed on RNase A and RNase B by microwave irradiating with Lyse-It more than one time at a higher power and time then previously tested or used. The higher microwave power and times were used in order to determine the conditions where 0% of the activity remained (i.e. the enzyme “kill” settings). DNase I only required 50% microwave power for 60 seconds of irradiation (27 kJ) to inactivate the nuclease as discussed previously. For RNase A and RNase B, three irradiations with Lyse-It at 70% microwave power for 105 seconds, a total energy subjected to the sample of 198.45 kJ, resulted in more than 99% inactivation of both nucleases (Fig 11). The rates from completely active nuclease compared to the “killed” inactive nucleases can be seen in Table 3. It is important to note that in particular for DNase I, even though the nuclease was completely inactive at 50% microwave power for 60 seconds, genomic DNA has shown to be still viable for detection and amplification through qPCR. [14]

**Table 3:** Nuclease inactivation studies.

*Nuclease*	Pre	“Kill”
	*Rate (Fluorescent Intensity per Second)*	
*20 pM RNase A*	322.43 ± 11.94	4.94 ± 0.21 (1.4%)
*49 pM RNase B*	477.04 ± 24.25	3.34 ± 2.22 (0.7%)
*10.5 nM DNase I*	215.85 ± 9.54	0.50 ± 0.10 (0.2%)

**Fig 12:**
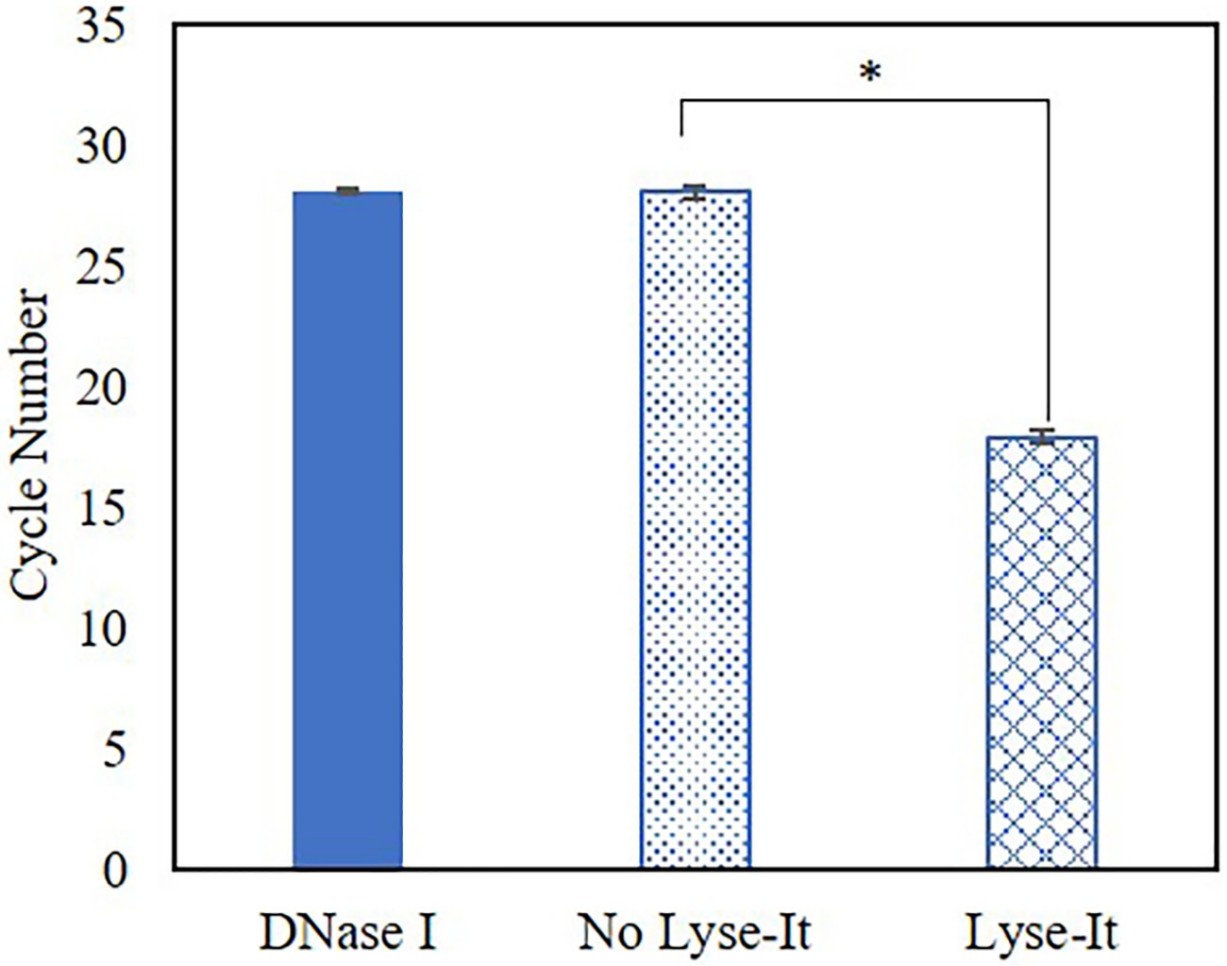
qPCR cycle number for amplified *V*. *cholerae* DNA before and after a 10-minute exposure to DNase I without microwave irradiation (DNase I), after 50% power 60 seconds standard microwave irradiation (No Lyse-It) and after 50% power, 60 seconds with Lyse-It (Lyse-It). A decrease in cycle number is seen with Lyse-It because the *V*. *cholerae* DNA is intact as compared to being chewed-up by intact DNase I. * = p = 0.001.

To demonstrate that DNase I is significantly inhibited by microwave irradiation with Lyse-It, qPCR was performed where *V*. *cholerae* DNA was subjected to stock intact DNase I, DNase I that was microwave irradiated for 60 seconds at 50% power without Lyse-It and then with Lyse-It. 0.5U of DNase I no microwave irradiation, with irradiation without Lyse-It or with Lyse-It was incubated with 1μg of *V*. *cholerae* DNA for 10 minutes. After 10 minutes the reaction was stopped and qPCR was performed. It was found that stock DNase I and DNase I that was microwave irradiated without Lyse-It broke down the *V*. *cholerae* DNA, thus the high cycle number (Fig 12). However, the cycle number significantly dropped when microwave irradiated DNase I with Lyse-It was incubated with *V*. *cholerae* DNA indicating that the DNase I is significantly inhibited from being able to break down the DNA.

## Discussion

In diagnostic settings like those that involve downstream manipulation of nucleic acid or other intracellular components, it is important that there are as few contaminants as possible and that the intracellular component of interest for detection is retained. Most sample preparation kits that are available remove contaminants like nucleases that can interfere with detection platforms such as qPCR.[29] However, these kits can be time consuming to use and expensive. Herein, we have demonstrated that Lyse-It is a rapid and cost-effective way of fragmenting and decreasing the activity of RNA and DNA nucleases. A cost analysis of Lyse-It compared to bead-beating, vortex, and protein saver cards has been reported in a recent work by Santaus et.al.[30]

We have shown in previous work that Lyse-It increased the overall temperature and electromagnetic energy to samples allowing for rapid cellular lysis and subsequent intracellular release followed by fragmentation and degradation of DNA and proteins.[12, 14] Herein, we have additionally shown that Lyse-It is also effective at fragmenting and inactivating RNase A, RNase B, and DNase I. SDS PAGE studies followed by Bioanalyzer mini-gel analysis demonstrated that nucleases were susceptible to thermal degradation and inactivation although this temperature dependence is not the dominant fragmentation mechanism associated with Lyse-It.[23, 24]

Though the use of SDS PAGE was beneficial to see the size of the nucleases, it was not sufficient to determine if nuclease fragmentation was occurring or moreover to rationale the reduction in nuclease activity. From the gels, it was determined that buffer concentration, when nucleases were conventionally heated, did not affect the ability of Oriole stain to bind to the nuclease. However, there were some statistical deviations in the band intensities as microwave power or irradiation time increased.

Following analysis of SDS PAGE band intensity, the 2100 Bioanalyzer was used to determine if nuclease fragmentation occurred following various tested conditions. It was found for all nucleases that as the oxygen content increased, the number of fragments also increased. Additionally, as microwave power or irradiation time increased, the number of fragments increased. Further analysis of the fragments was performed to determined weighted size and standard error. As microwave power and time increased, the weighted size of the nucleases decreased and the standard error of the weighted size increased. This is indicative of the nucleases being fragmented down to smaller and then smaller sizes, where the fragments were of a variety of sizes. As mentioned earlier, simple microwave heating and a temperature increase, does not appear to be the dominant mechanism behind DNA, RNA, and protein fragmentation.[23, 24] Previous studies have shown a significant amount of reactive oxygen species (ROS) is released during Lyse-It. We therefore speculated that nuclease fragmentation and subsequent inactivation is due to ROS when using Lyse-It.

Further investigation using conventional heating, standard microwave irradiation, and Lyse-It were performed. It was determined that out of the three methods investigated that Lyse-It had a much stronger effect on nuclease inactivation. Conventional heating until higher temperatures did not significantly affect the overall nuclease activity and standard microwave irradiation only decreased the activity about 40%. Remarkably, Lyse-It decreased the activity over 70% with a microwave irradiation power and time of 50%, 60 seconds. Additionally, we noticed, though not completely understood, that HEPES has a protective property against nuclease activity degradation.

In closing, Lyse-It can serve as a tunable platform for nuclease degradation and inactivity, which can be beneficial increasing the accuracy of platforms like qPCR.

## Supporting information

S1 Fig Standard Lyse-It slide with a sample chamber prior to purging (A). Lyse-It purging system with a sample chamber and safety lid, with both a gas-in and gas-out needle.
(TIF)

S2 Fig Simplified RNaseAlert and DNaseAlert assay systems. Prior to the addition of nuclease, the fluorophore exhibits low fluorescence. Upon addition of nuclease and subsequent cutting of the fluorophore/quencher system, the fluorophore exhibits an increase in fluorescence which can be readily monitored using fluorescence kinetics, i.e. by monitoring the fluorescent intensity versus time.
(TIF)

S3 Fig Normalized SDS PAGE band intensity of conventionally heated (60°C) of RNase A, RNase B, and DNase I in 2mM Tris-EDTA and HEPES buffers with representative SDS PAGE **(A)** RNase A, **(B)** DNase I, and **(C)** RNase B. In all cases, no significant change in band intensity was observed. (p>> 0.05) **(D)** RNase A in Tris-EDTA and HEPES buffers SDS PAGE. L: Ladder, P: RNase A Pre, DI: Nuclease in DI and conventionally heated, 1–5: decreasing Tris-EDTA buffer concentration, 6–10: decreasing HEPES buffer concentration. **(E)** RNase B conventionally heated in 1 mM Tris-EDTA or HEPES buffers. L: Ladder, T: RNase B in Tris-EDTA no heating, 1–5: 40–80°C, H: RNase B in HEPES no heating, 6–10: 40–80°C.
(TIF)

S4 Fig RNase A in Tris-EDTA, HEPES, and DI water with respective statistical analysis for SDS PAGE band intensities. **(A)** Microwave Power Comparison of RNase A in Tris-EDTA, HEPES, or DI Water irradiated for 30 seconds, **(B)** Statistical analysis of RNase A in the three buffers as compared to 30% or 50% power, **(C)** Statistical analysis of the change in band intensity versus microwave power of RNase A in the three buffers. (D) RNase A in Tris-EDTA, HEPES, or DI SDS PAGE, P# = RNase A in buffer no microwave irradiation, 1: 30% Power, 2: 50% Power.
(TIF)

S5 Fig SDS PAGE band intensities for RNase B in Tris-EDTA, HEPES, or DI water with respective statistical analysis. **(A)** Microwave power comparison of RNase B in Tris-EDTA, HEPES, or DI Water irradiated for 30 seconds, **(B)** Statistical analysis of RNase B in the three buffers as compared to 30% or 50% power, **(C)** Statistical analysis of the change in microwave power of RNase B in the three buffers.
(TIF)

S6 Fig Fluorescent intensity versus time for DNase I in Tris-EDTA, HEPES, or DI water with respective statistical analysis. **(A)** Microwave Power Comparison of DNase I in Tris-EDTA, HEPES, or DI Water, irradiated for 30 seconds **(B)** Statistical analysis of DNase I in the three buffers as compared to 30% or 50% power, **(C)** Statistical analysis of DNase I in the three buffers.
(TIF)

S7 Fig Representative SDS-PAGE gel of RNase B showing gel abnormalities (curved gel and non-compressed bands in the white circles) attributing to the increase in band intensity as microwave irradiation time increased shown in Fig 1 for RNase B. (JPG)

S1 Table Fragment sizes determined post Lyse-It after purging with argon, air, or oxygen. Increasing oxygen concentration results in an increase in number of peaks due to fragmentation.
(DOCX)

S2 Table Increasing microwave power results in an increase in number of peaks due to fragmentation. (DOCX)

S3 Table Increasing microwave irradiation time results in an increase in number of peaks due to fragmentation. (DOCX)

S4 Table RNase A and DNase I rates and percentage still active post conventional heating for 1 minute between 40°C and 80°C. (DOCX)

S5 Table Nuclease rates and percentage still active post Lyse-It in 1 mM buffers at 50% power, 60 seconds. (Pre = no irradiation).
(DOCX)

S6 Table Nuclease rates and percentage still active with and without Lyse-It in DI water at 50% power, 60 seconds. (Pre = no irradiation).
(DOCX)

S7 Table Nuclease rates and percentage still active post Lyse-It at 30% and 50% power, varying the time. (Pre = no irradiation).
(DOCX)
