# Supplementary figures and images for: Effects of Lyse-It on endonuclease fragmentation, function and activity
Source: PLoS One. 2019 Sep 30;14(9):e0223008. doi: 10.1371/journal.pone.0223008 (PMC6768537; doi:10.1371/journal.pone.0223008)

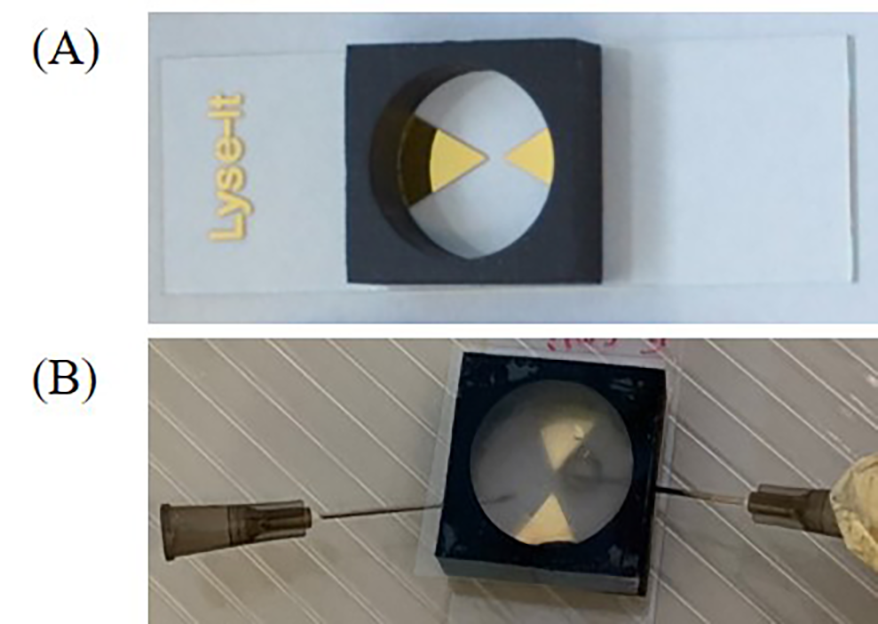

Supplement: S1 Fig — Lyse-It purging system with a sample chamber and safety lid, with both a gas-in and gas-out needle. (TIF) [file pone.0223008.s001.tif]

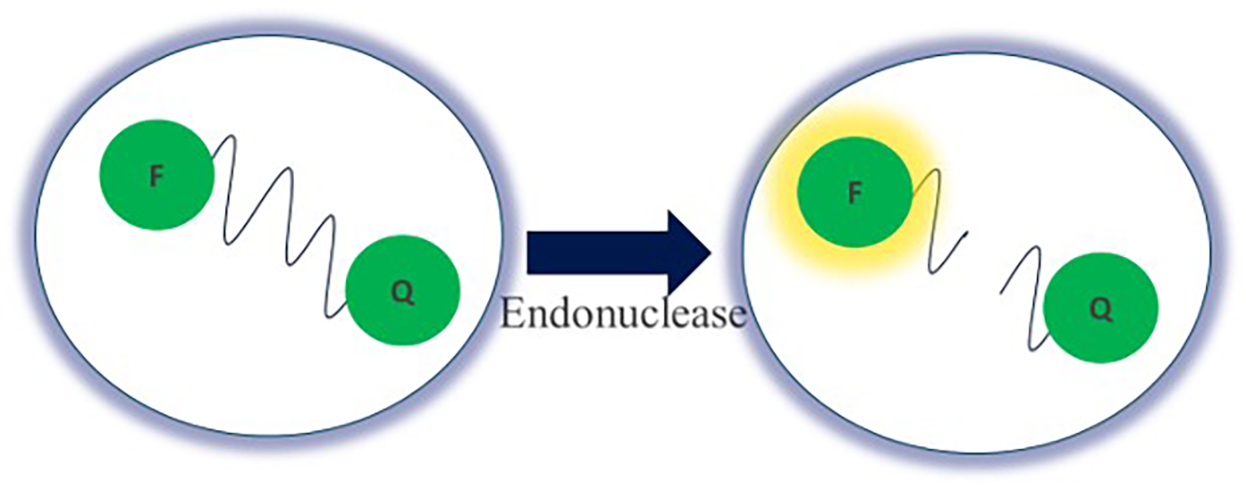

Supplement: S2 Fig — Prior to the addition of nuclease, the fluorophore exhibits low fluorescence. Upon addition of nuclease and subsequent cutting of the fluorophore/quencher system, the fluorophore exhibits an increase in fluorescence which can be readily monitored using fluorescence kinetics, i.e. by monitoring the fluorescent intensity versus time. (TIF) [file pone.0223008.s002.tif]

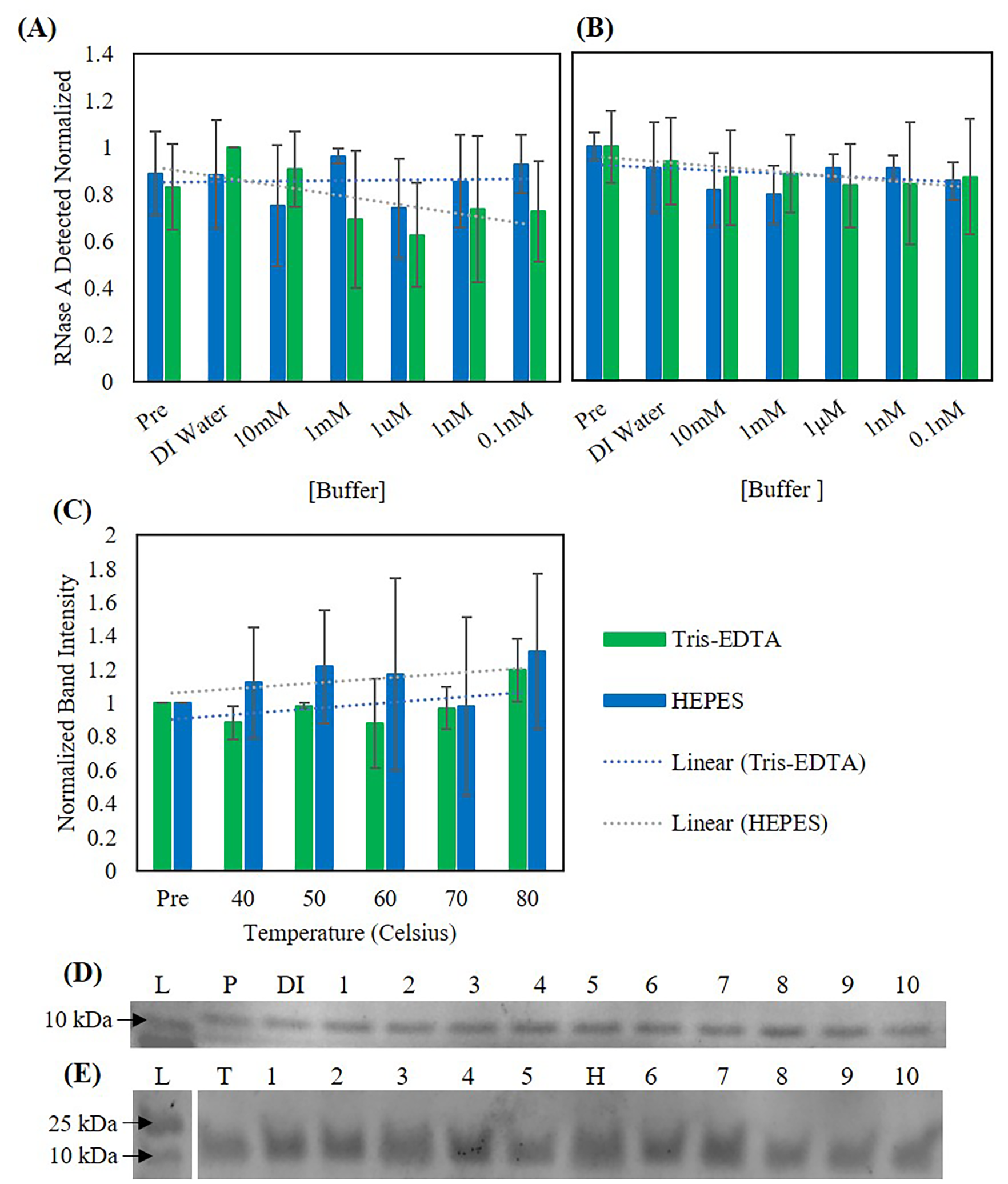

Supplement: S3 Fig — Normalized SDS PAGE band intensity of conventionally heated (60°C) of RNase A, RNase B, and DNase I in 2mM Tris-EDTA and HEPES buffers with representative SDS PAGE (A) RNase A, (B) DNase I, and (C) RNase B. In all cases, no significant change in band intensity was observed. (p>> 0.05) (D) RNase A in Tris-EDTA and HEPES buffers SDS PAGE. L: Ladder, P: RNase A Pre, DI: Nuclease in DI and conventionally heated, 1–5: decreasing Tris-EDTA buffer concentration, 6–10: decreasing HEPES buffer concentration. (E) RNase B conventionally heated in 1 mM Tris-EDTA or HEPES buffers. L: Ladder, T: RNase B in Tris-EDTA no heating, 1–5: 40–80°C, H: RNase B in HEPES no heating, 6–10: 40–80°C. (TIF) [file pone.0223008.s003.tif]

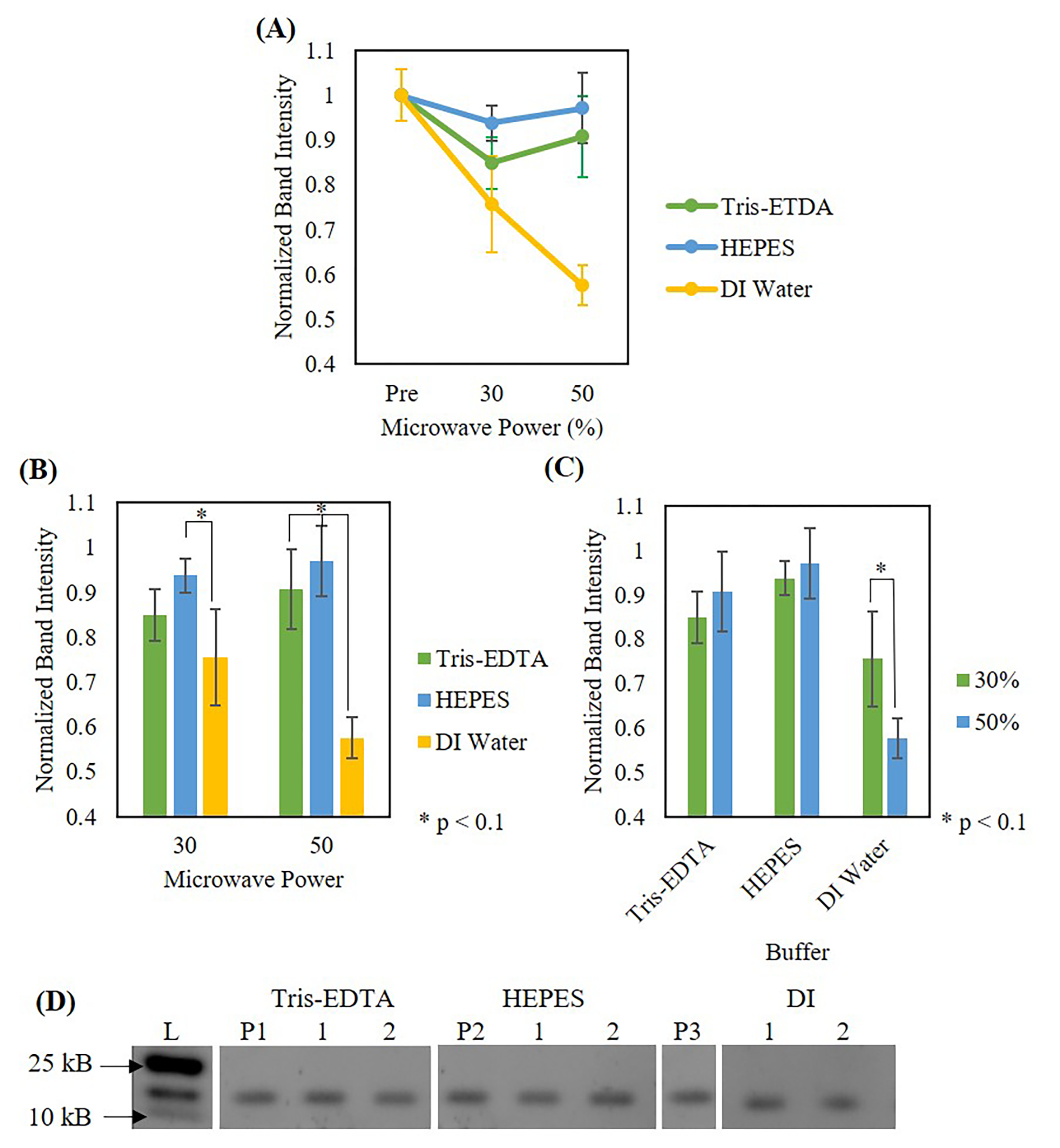

Supplement: S4 Fig — (A) Microwave Power Comparison of RNase A in Tris-EDTA, HEPES, or DI Water irradiated for 30 seconds, (B) Statistical analysis of RNase A in the three buffers as compared to 30% or 50% power, (C) Statistical analysis of the change in band intensity versus microwave power of RNase A in the three buffers. (D) RNase A in Tris-EDTA, HEPES, or DI SDS PAGE, P# = RNase A in buffer no microwave irradiation, 1: 30% Power, 2: 50% Power. (TIF) [file pone.0223008.s004.tif]

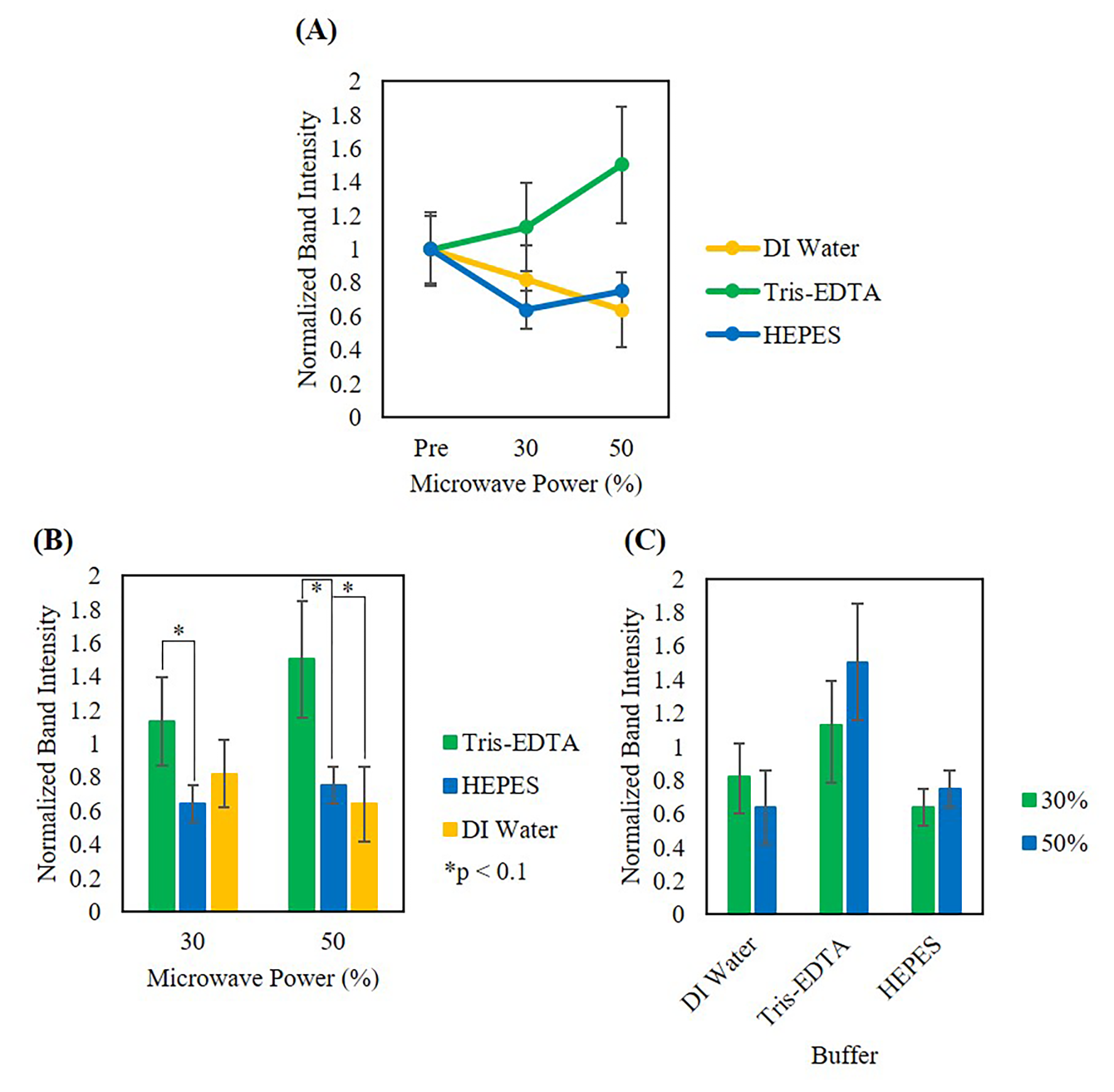

Supplement: S5 Fig — (A) Microwave power comparison of RNase B in Tris-EDTA, HEPES, or DI Water irradiated for 30 seconds, (B) Statistical analysis of RNase B in the three buffers as compared to 30% or 50% power, (C) Statistical analysis of the change in microwave power of RNase B in the three buffers. (TIF) [file pone.0223008.s005.tif]

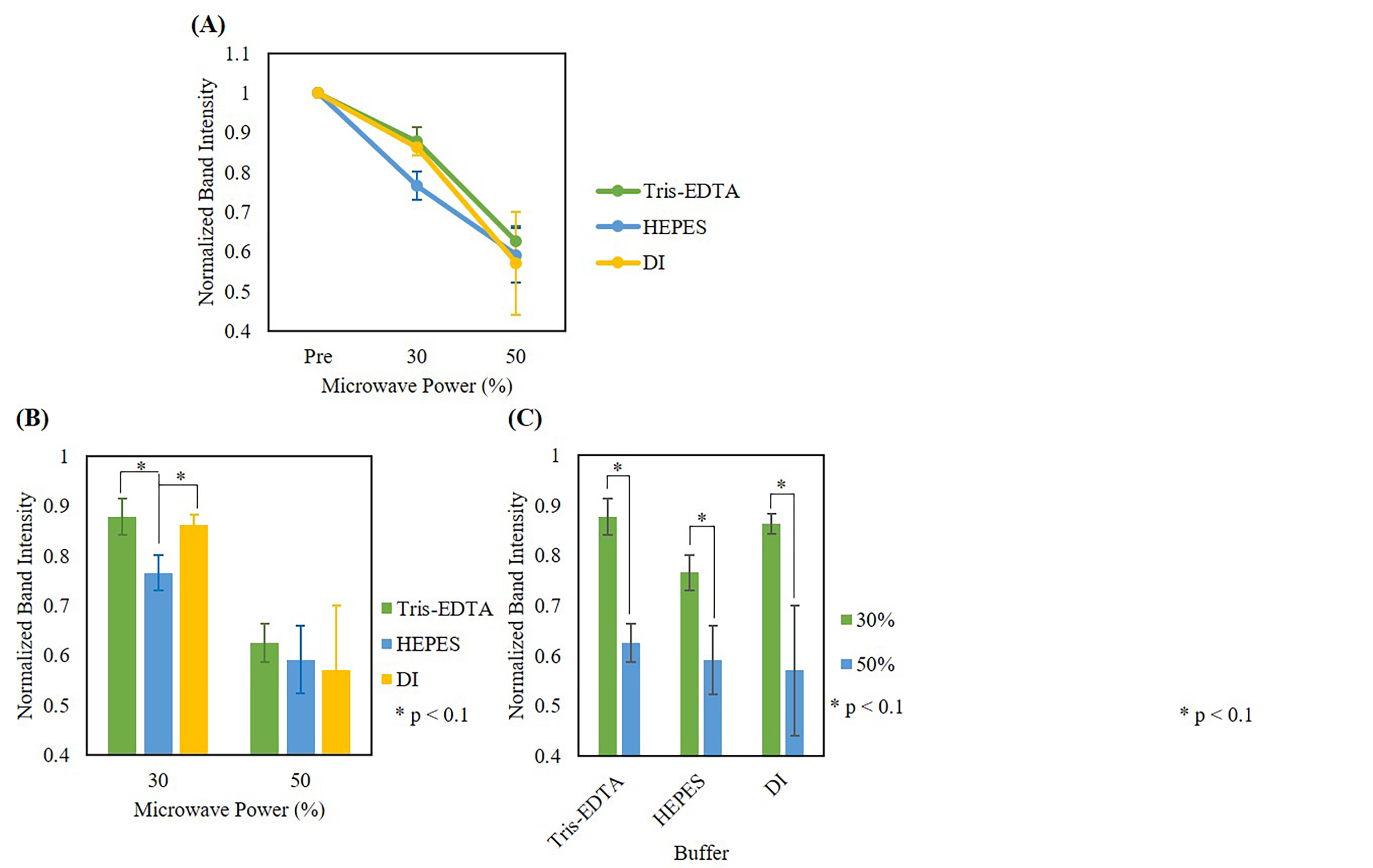

Supplement: S6 Fig — (A) Microwave Power Comparison of DNase I in Tris-EDTA, HEPES, or DI Water, irradiated for 30 seconds (B) Statistical analysis of DNase I in the three buffers as compared to 30% or 50% power, (C) Statistical analysis of DNase I in the three buffers. (TIF) [file pone.0223008.s006.tif]

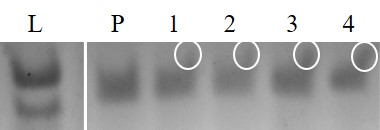

Supplement: S7 Fig — (JPG) [file pone.0223008.s007.jpg]
