# Supplementary material for: Effects of Lyse-It on endonuclease fragmentation, function and activity
Source: PLoS One. 2019 Sep 30;14(9):e0223008. doi: 10.1371/journal.pone.0223008 (PMC6768537; doi:10.1371/journal.pone.0223008)
Supplement: S1 Table — Increasing oxygen concentration results in an increase in number of peaks due to fragmentation. (DOCX) [file pone.0223008.s008.docx]

| kDa | 5 to 15 | 16 to 30 | 31 to 45 | Total Peaks |
| --- | --- | --- | --- | --- |
| **RNase A (13.7kDa)** | | | | |
| **Pre** | 0 | 1 | 0 | **1** |
| **Argon** | 0 | 1 | 0 | **1** |
| **Air** | 0 | 2 | 0 | **2** |
| **Oxygen** | 3 | 2 | 0 | **5** |
| **RNase B (14.8 kDa)** | | | | |
| **Pre** | 0 | 1 | 0 | **1** |
| **Argon** | 0 | 1 | 2 | **3** |
| **Air** | 1 | 0 | 0 | **3** |
| **Oxygen** | 1 | 1 | 0 | **5** |
| **DNase I (approx. 31 kDa)** | | | | |
| **Pre** | 0 | 0 | 1 | **1** |
| **Argon** | 1 | 0 | 1 | **2** |
| **Air** | 2 | 0 | 1 | **3** |
| **Oxygen** | 2 | 2 | 1 | **5** |

**S1 Table:** Fragment sizes determined post Lyse-It^®^ after purging with argon, air, or oxygen. Increasing oxygen concentration results in an increase in number of peaks due to fragmentation.
