# Supplementary material for: Effects of Lyse-It on endonuclease fragmentation, function and activity
Source: PLoS One. 2019 Sep 30;14(9):e0223008. doi: 10.1371/journal.pone.0223008 (PMC6768537; doi:10.1371/journal.pone.0223008)
Supplement: S2 Table — (DOCX) [file pone.0223008.s009.docx]

| kDa | 5 - 15 | 16 - 30 | 31 - 45 | 45 - 60 | 61+ | Total Peaks | |
| --- | --- | --- | --- | --- | --- | --- | --- |
| **RNase A (13.7kDa)** | | | | | | |  |
| **Pre** | 0 | 1 | 0 | 0 | 0 | **1** | |
| **30% Power** | 0 | 1 | 0 | 0 | 0 | **1** | |
| **50% Power** | 0 | 4 | 1 | 0 | 0 | **5** | |
| **RNase B (14.8 kDa)** | | | | | | |  |
| **Pre** | 0 | 1 | 0 | 0 | 0 | **1** | |
| **30% Power** | 0 | 1 | 0 | 0 | 0 | **1** | |
| **50% Power** | 7 | 7 | 0 | 2 | 0 | **16** | |
| **DNase I (approx. 31 kDa)** | | | | | | |  |
| **Pre** | 0 | 0 | 1 | 0 | 0 | **1** | |
| **30% Power** | 5 | 0 | 1 | 1 | 1 | **8** | |
| **50% Power** | 2 | 1 | 5 | 5 | 6 | **19** | |

**S2 Table:** Increasing microwave power results in an increase in number of peaks due to fragmentation.
