# Supplementary material for: Effects of Lyse-It on endonuclease fragmentation, function and activity
Source: PLoS One. 2019 Sep 30;14(9):e0223008. doi: 10.1371/journal.pone.0223008 (PMC6768537; doi:10.1371/journal.pone.0223008)
Supplement: S3 Table — (DOCX) [file pone.0223008.s010.docx]

| kDa | 5 - 15 | 16 - 30 | 31 - 45 | Total Peaks |
| --- | --- | --- | --- | --- |
| **RNase A (13.7kDa) 30% Power** | | | | |
| **Pre** | 0 | 1 | 0 | **1** |
| **30 seconds** | 0 | 1 | 0 | **1** |
| **45 seconds** | 0 | 1 | 0 | **1** |
| **60 seconds** | 0 | 1 | 0 | **1** |
| **135 seconds** | 0 | 1 | 0 | **1** |
| **RNase B (14.8 kDa) 30% Power** | | | | |
| **Pre** | 0 | 1 | 0 | **1** |
| **30 seconds** | 0 | 1 | 2 | **3** |
| **45 seconds** | 0 | 1 | 2 | **3** |
| **60 seconds** | 0 | 2 | 1 | **3** |
| **135 seconds** | 1 | 6 | 1 | **8** |
| **DNase I (approx. 31 kDa) 30% Power** | | | | |
| **Pre** | 0 | 0 | 1 | **1** |
| **30 seconds** | 0 | 0 | 1 | **1** |
| **45 seconds** | 1 | 0 | 1 | **2** |
| **60 seconds** | 2 | 0 | 1 | **3** |
| **135 seconds** | 3 | 1 | 1 | **5** |

**S3 Table:** Increasing microwave irradiation time results in an increase in number of peaks due to fragmentation.
