# Supplementary material for: Effects of Lyse-It on endonuclease fragmentation, function and activity
Source: PLoS One. 2019 Sep 30;14(9):e0223008. doi: 10.1371/journal.pone.0223008 (PMC6768537; doi:10.1371/journal.pone.0223008)
Supplement: S4 Table — (DOCX) [file pone.0223008.s011.docx]

| **Conventional Heating – Temperature (˚C)** | **Rate (Fluorescent Intensity per Second)** | **Nuclease Percentage**  **Still Active** |
| --- | --- | --- |
| **RNase A** | | |
| Pre (RT) | 247.29 ± 13.56 | 100% |
| 40 | 114.96 ± 8.92 | 46.5% |
| 50 | 126.28 ± 0.48 | 51.1% |
| 60 | 124.80 ± 2.21 | 50.5% |
| 70 | 144.76 ± 4.84 | 58.5% |
| 80 | 138.76 ± 3.64 | 56.1% |
| **DNase I** | | |
| Pre (RT) | 406.8 ± 24.0 | 100% |
| 40 | 324.8 ± 73.8 | 100% |
| 50 | 242.4 ± 30.2 | 59.6% |
| 60 | 270.5 ± 41.0 | 66.5% |
| 70 | 127.5 ± 5.7 | 31.3% |
| 80 | 7.9 ± 3.3 | 1.9% |

**S4 Table**: RNase A and DNase I rates and percentage still active post conventional heating for 1 minute between 40˚C and 80˚C.
