# Supplementary material for: Effects of Lyse-It on endonuclease fragmentation, function and activity
Source: PLoS One. 2019 Sep 30;14(9):e0223008. doi: 10.1371/journal.pone.0223008 (PMC6768537; doi:10.1371/journal.pone.0223008)
Supplement: S5 Table — (Pre = no irradiation). (DOCX) [file pone.0223008.s012.docx]

| **Buffer** | **Rate (Fluorescent intensity per second)** | **Nuclease Percentage Still Active** |
| --- | --- | --- |
| **RNase A** | | |
| Pre (20pM) | 119.6 ± 3.4 (100%) | 100% |
| 20 pM in Tris-EDTA | 78.1 ± 1.0 (65%) | 65% |
| 20 pM in HEPES | 97.5 ± 2.5 (82%) | 82% |
| 20 pM in DI Water | 55.2 ± 1.7 (46%) | 46% |
| **RNase B** | | |
| Pre (46pM) | 109.2 ± 4.2 | 100% |
| 46 pM in Tris-EDTA | 104.1 ± 1.3 (95%) | 95% |
| 46 pM in HEPES | 109.6 ± 5.6 (100%) | 100% |
| 46 pM in DI Water | 84.9 ± 1.5 (78%) | 78% |
| **DNase I** | | |
| Pre (10.5nM) | 261.81 ± 10.22 | 100% |
| 10.5 nM in Tris-EDTA | 0 | 0% |
| 10.5 nM in HEPES | 5.33 ± 1.51 | 2% |
| 10.5 nM in DI Water | 0 | 0% |

**S5 Table**: Nuclease rates and percentage still active post Lyse-It^®^ in 1 mM buffers at 50% power, 60 seconds. (Pre = no irradiation)
