# Supplementary material for: Effects of Lyse-It on endonuclease fragmentation, function and activity
Source: PLoS One. 2019 Sep 30;14(9):e0223008. doi: 10.1371/journal.pone.0223008 (PMC6768537; doi:10.1371/journal.pone.0223008)
Supplement: S6 Table — (Pre = no irradiation). (DOCX) [file pone.0223008.s013.docx]

| **Nuclease Concentration and No Lyse-It^®^ vs. Lyse-It^®^** | **Rate (Fluorescent Intensity per Second)** | **Nuclease Percentage**  **Still Active** |
| --- | --- | --- |
| **RNase A** | | |
| Pre (20.1pM) | 211.0 ± 7.6 | 100% |
| Pre (2.01pM) | 42.9 ± 1.0 | 20% |
| Pre (0.201 pm) | 3.2 ± 0.5 | 1.5% |
| 20.1 pM 50%, 60s Lyse-It^®^ | 55.2 ± 1.7 | 26% |
| 20.1 pM 50%, 60s No Lyse-It^®^ | 129.7 ± 5.3 | 61% |
| **RNase B** | | |
| Pre (46pM) | 452.0 ± 24.53 | 100% |
| Pre (4.6pM) | 136.4 ± 0.01 | 30% |
| Pre (0.46 pm) | 14.7 ± 0.55 | 3% |
| 46 pM 50%, 60s Lyse-It^®^ | 133.9 ± 9.67 | 30% |
| 46 pM 50%, 60s No Lyse-It^®^ | 303.7 ± 10.78 | 67% |
| **DNase I** | | |
| Pre (10.5nM) | 164.66 ± 3.19 | 100% |
| Pre (1.05nM) | 13.08 ± 0.46 | 8% |
| Pre (0.105nM) | 0.66 ± 0.64 | 0.4% |
| 10.5 nM 50%, 60s Lyse-It^®^ | 0.56 ± 0.55 | 0.3% |
| 10.5 nM 50%, 60s No Lyse-It^®^ | 102.19 ± 4.69 | 62% |

**S6 Table**: Nuclease rates and percentage still active with and without Lyse-It^®^ in DI water at 50% power, 60 seconds. (Pre = no irradiation)
