# Supplementary material for: Effects of Lyse-It on endonuclease fragmentation, function and activity
Source: PLoS One. 2019 Sep 30;14(9):e0223008. doi: 10.1371/journal.pone.0223008 (PMC6768537; doi:10.1371/journal.pone.0223008)
Supplement: S7 Table — (Pre = no irradiation). (DOCX) [file pone.0223008.s014.docx]

| **Nuclease Irradiation Time (seconds)** | **30% Microwave Power Rate (Fluorescence Intensity per Second)** | **50% Microwave Power Rate (Fluorescence Intensity per Second)** |
| --- | --- | --- |
| **RNase A** | | |
| Pre | 97.2 ± 1.5 (100%) | |
| 30 seconds | 46.9 ± 0.8 (48%) | 33.8 ± 1.0 (35%) |
| 60 seconds | 52.4 ± 0.8 (54%) | 7.3 ± 0.6 (8%) |
| 90 seconds | 8.9 ± 0.4 (9%) | 7.1 ± 0.5 (7%) |
| **DNase I** | | |
| Pre | 122.58 ± 3.9-0 (100%) | |
| 30 seconds | 94.55 ± 0.23 (77%) | --- |
| 60 seconds | 28.31 ± 2.23 (23%) | --- |
| 90 seconds | 3.27 ± 0.41 (3%) | --- |

**S7 Table**: Nuclease rates and percentage still active post Lyse-It^®^ at 30% and 50% power, varying the time. (Pre = no irradiation)
